# Ectopic Expression of *CDF3* Genes in Tomato Enhances Biomass Production and Yield under Salinity Stress Conditions

**DOI:** 10.3389/fpls.2017.00660

**Published:** 2017-05-03

**Authors:** Begoña Renau-Morata, Rosa V. Molina, Laura Carrillo, Jaime Cebolla-Cornejo, Manuel Sánchez-Perales, Stephan Pollmann, José Domínguez-Figueroa, Alba R. Corrales, Jaume Flexas, Jesús Vicente-Carbajosa, Joaquín Medina, Sergio G. Nebauer

**Affiliations:** ^1^Área de Fisiología Vegetal, Universitat Politècnica de ValènciaValència, Spain; ^2^Centro de Biotecnología y Genómica de PlantasMadrid, Spain; ^3^Departamento de Biotecnología, Universitat Politècnica de ValènciaValència, Spain; ^4^Departamento de Biología, Universitat de les Illes BalearsPalma, Spain

**Keywords:** CDF, tomato, photosynthesis, abiotic stress, crop yield, C/N metabolism, transcriptome

## Abstract

Cycling Dof Factor (CDF) transcription factors (TFs) are involved in multiple processes related to plant growth and development. A member of this family, CDF3, has recently been linked in Arabidopsis to the regulation of primary metabolism and abiotic stress responses, but its role in crop production under stress is still unknown. In this study, we characterized tomato plants overexpressing the *CDF3* genes from Arabidopsis and tomato and analyzed their effects on growth and yield under salinity, additionally gaining deeper insights into the molecular function of these TFs. Our results provide evidence for higher biomass production and yield in the *35S::AtCDF3* and *35S::SlCDF3* plants, likely due to a higher photosynthetic capacity resulting in increased sucrose availability. Transcriptome analysis revealed that *CDF3* genes regulate a set of genes involved in redox homeostasis, photosynthesis performance and primary metabolism that lead to enhanced biomass production. Consistently, metabolomic profiling revealed that CDF3 evokes changes in the primary metabolism triggering enhanced nitrogen assimilation, and disclosed that the amount of some protective metabolites including sucrose, GABA and asparagine were higher in vegetative tissues of *CDF3* overexpressing plants. Altogether these changes improved performance of *35S::AtCDF3* and *35S::SlCDF3* plants under salinity conditions. Moreover, the overexpression of *CDF3* genes modified organic acid and sugar content in fruits, improving variables related to flavor perception and fruit quality. Overall, our results associate the CDF3 TF with a role in the control of growth and C/N metabolism, and highlight that overexpression of *CDF3* genes can substantially improve plant yield.

## Introduction

In the twentieth century, enhancement of crop production was achieved through improving agricultural practices to optimize photoassimilate production, and to increase the harvest index by altering photoassimilate partitioning. However, these strategies appear to have peaked in many crops, and have been achieved without increasing total biomass (Paul and Foyer, 2001). Moreover, the impact of climate change, increasing urbanization, salinity of soils and aquifers, and desertification, limit food production and present an unprecedented challenge (Godfray et al., 2010). In this context, the demand for higher productivity under prevailing conditions requires improving photosynthetic efficiency and biomass production, as well as increased crop tolerance to abiotic factors, to facilitate the usage of marginal lands and partially salinized waters.

Increased leaf photosynthesis is closely associated with increased yields when assaying the growth of a genotype under diverse environmental conditions or a narrow range of germplasm within a certain species (Long et al., 2006). Six potential routes to improve photosynthetic efficiency, which ranged from altered canopy architecture to the improved regeneration of the acceptor molecule for CO_2_, have been explored by Long et al. (2015). In relation to the light reactions of photosynthesis, the extension of the usable spectrum of light and the improvement of heat dissipation pathways have been proposed as biotechnological targets. Furthermore, the engineering of CO_2_ concentration pathways, improved Rubisco forms, photorespiration bypasses and the optimization of RuBP regeneration systems have been suggested for the dark reactions of photosynthesis. These authors concluded that the changes to improve the efficiency of converting intercepted light into biomass and, therefore, yield, could be achieved by transgenic technology. Nonetheless, transferring different individual genes of interest is not practical given the number of enzymes involved in a metabolic pathway. Several recent studies have indicated the possibility of using transcription factors (TFs), which control the expression of several genes, to enhance photosynthesis and biomass production (Yanagisawa et al., 2004; Saibo et al., 2009).

Drought, salinity and low temperature are environmental constraints that diminish photosynthetic efficiency and adversely affect plant growth and productivity. These unfavorable growth conditions impose water deficit, and stomatal closure as well as lower CO_2_ diffusion are the earliest responses (Tezara et al., 1999; Flexas and Medrano, 2002; Saibo et al., 2009). Furthermore, membranes may become disorganized, proteins may undergo loss of activity or be denatured, and oxidative damage often occurs. As a result, inhibition of photosynthesis, a metabolic dysfunction, and damage to cellular structures contribute to stunted growth (Krasensky and Jonak, 2012). Premature senescence in photosynthetic source tissues lowers the number and limits the growth of harvestable sink organs (Albacete et al., 2014). Plants respond to abiotic stresses by means of biochemical and physiological adaptations, which involve changes in gene expression, including genes that encode the enzymes responsible for the biosynthesis of osmolytes, detoxification enzymes, transport proteins, late-embryogenesis-abundant proteins or genes that regulate phytohormone levels (Pareek et al., 2010). In addition, plants have evolved specific strategies to maintain photosynthetic efficiency. This could involve several processes at different levels, including CO_2_ diffusion, light harvesting, redox homeostasis, carbon assimilation and carbohydrate metabolism (Abreu et al., 2013), and changes in the expression of many genes (Saibo et al., 2009; Abreu et al., 2013).

Altogether, this differential gene expression is regulated by specific TFs (Yamaguchi-Shinozaki and Shinozaki, 2006; Saibo et al., 2009) and, in line with this, altering the expression of these TFs can increase crop tolerance (Varshney et al., 2011; Datta et al., 2012; Hichri et al., 2016). The observed effects on photosynthesis efficiency or photosynthetic machinery are normally positive in these tolerant plants, as indicated by Saibo et al. (2009). However, these results must be interpreted with caution because abiotic stress tolerance has been evaluated mostly under laboratory conditions and very little is known about plant responses to adverse field conditions. Very few cases have been reported in which physiological research has led to improve crop cultivars to produce increased yields (Sinclair et al., 2004; Datta et al., 2012). The work of Gupta et al. (2012) in millet has reported greater photosynthetic efficiency, and also better growth, yield and crude grain protein content by overexpressing the DOF1 gene. Improved nitrogen assimilation and growth under low-nitrogen conditions in plants of Arabidopsis and rice that overexpress maize DOF1 have been previously described (Yanagisawa et al., 2004; Kurai et al., 2011). DOF TFs are of particular interest if we take into account the relation between their higher expression and increased photosynthetic rate, but also improved agronomic yield.

DNA binding with One Finger (DOF) proteins are a group of plant-specific TFs that contain a highly conserved domain that binds specifically to a 5′-T/AAAAG-3′ sequence motif in the promoters of direct target genes (Yanagisawa and Schmidt, 1999). DOF proteins have been reported to be involved in the control of very different growth and development processes in plants (Yanagisawa, 2002, 2004). The characterization of five *DOF* genes from tomato, homologous to Arabidopsis Cycling DOF Factors (CDFs), has been recently published (Corrales et al., 2014). *SlCDF1–5* genes were differentially induced in response to osmotic, salt, heat, and low-temperature stress. The Arabidopsis plants that overexpressed tomato orthologous genes *SlCDF1* or *SlCDF3* showed increased drought and salt tolerance. The metabolic analyses of these plants presented higher levels of sucrose and different amino acids, which indicates increased nitrogen assimilation, as reported previously for other DOF TFs (Yanagisawa et al., 2004). A multifaceted role for the Arabidopsis *AtCDF3* gene in stress responses and development in Arabidopsis has also been established (Corrales et al., 2017). In this work, the authors revealed that AtCDF3 regulates a set of genes involved in cellular osmoprotection and oxidative stress, including key stress tolerance TFs like CBFs, DREB2A and ZAT12. In addition, Fornara et al. (2015) reported that Arabidopsis CDFs promote plant growth through the action of PIF4 and IAA29 transcription factors. Together all these data highlight the impact of CDFs on plant development and on the interaction with environmental cues.

Based on these results it appeared tempting to us to investigate the possibility to use the *CDF* genes as a biotechnological tool in tomato breeding. With this aim in mind, we generated tomato plants overexpressing the *CDF3* genes from tomato and Arabidopsis to address the question whether by this it is possible to increase salt tolerance and improve yield. Here we demonstrate that the overexpression of *AtCDF3* or the orthologous tomato gene *SlCDF3* in tomato plants increases photosynthetic rate and biomass production, resulting in higher yields. While non-transformed plants suffered a considerable performance decrease under salinity stress conditions, photosynthesis and yield remained significantly higher in the *35S::CDF3* tomato plants. In addition to the improved performance under salinity (Corrales et al., 2014, 2017), our data suggest that the ectopic expression of *CDF3* improves the photosynthetic capacity which, in turn, leads to higher sucrose availability and changes in the plant's primary metabolism facilitating enhanced N assimilation, being the reason for the increased biomass production.

## Materials and methods

### Plant materials and growth conditions

Non-transformed (NT) *Solanum lycopersicum* cv. Moneymaker was used as the WT. Tomato seeds were germinated and cultured as described in Renau-Morata et al. (2014). Plants were grown in hydroponic culture with Hoagland no. 2 solution (Hoagland and Arnon, 1950) at 16/8 h light/dark photoperiod, 200 μmol m^−2^ s^−1^ light irradiance and 25/18°C light/dark.

For the greenhouse assays, imbibed seeds were germinated on a moistened mixture of peat moss and sand in growth chambers at 25/18°C and a 16/8 h photoperiod. Seedlings were transferred to 15 L pots that contained coconut coir fiber and were irrigated with Hoagland no. 2 nutrient solution.

### Constructs and tomato transformation

The ORFs of the *AtCDF3* (*Arabidopsis thaliana* Col-0) and *SlCDF3* (*Solanum lycopersicum* cv. Moneymaker) genes were amplified by PCR using cDNA as a template. They were cloned into a binary vector under the control of the CaMV35S promoter, followed at the 3′end by the nopaline synthase gene (NOS) terminator (Figure S1). The resultant plasmids were used to transform tomato plants cv. Moneymaker following the method described by Ellul et al. (2003). The seeds from the transformed plants were harvested and plated on selective medium and the kanamycin-resistant seedlings were transplanted to soil. The next generation seeds were subjected to a second round of selection to determine the homozygous lines.

### Salt stress assays

Thirty-day-old plants (three to four leaves) grown in hydroponic culture were used for the stress assays. Saline stress experiments were performed by adding sodium chloride at 75 mM to the nutrient solution (EC 7–8 dS m^−1^), as described in the standardized procedures by Renau-Morata et al. (2014). Tolerance was determined by measuring biomass and photosynthetic capacity after 15 days of treatment.

A second experiment was conducted under the above-described conditions. The behavior of the 30-day-old plants was compared for 15 days under the salinity and control conditions in growth chambers. Twelve plants were used for each genotype and treatment. For each treatment and genotype, plant material was pooled in three independent extracts (leaves of 4 plants per extract) of 45-day-old-plants, was immediately frozen (N_2_) after sampling and stored at −80°C until use. Sampling was performed at zeitgeber time 2 (ZT2). The transcriptomic analysis, and the metabolomic, hormones and mineral element determinations, were performed with the same plant material.

### Biomass quantification

The fresh weights of shoots and roots, leaf area and number of leaves were measured after 15 days of stress treatment (salt and low temperature conditions). Determinations were made on 10 different plants for each genotype and treatment. The dry weights of shoots and roots were measured after drying at 60°C for 48 h.

### Measurement of photosynthetic activity

The instantaneous values of net CO_2_ assimilation rate (A_N_, μmol m^−2^ s^−1^), stomatal conductance (g_s_, mol m^−2^ s^−1^), substomatal CO_2_ concentration (Ci, μmol mol^−1^) and transpiration rate (E, mmol m^−2^ s^−1^) were determined with an LI-6400 infrared gas analyser (LICOR Biosciences, Lincoln, USA). One measurement per plant was taken on the third or fourth leaf from the apex. Eight to ten different plants were used. The conditions in the measuring chamber were controlled at a flow rate of 500 mol s^−1^, a saturating PAR of 1,200 μmol m^−2^ s^−1^, 400 ppm CO_2_ and 60–70% relative humidity. Maximum photochemical efficiency (F_v_/F_m_) on the dark-adapted leaves was measured by a portable pulse amplitude modulation fluorometer (MINI PAM, Walz, Effeltrich, Germany).

After inducing steady-state photosynthesis, A_N_-C_i_ curves were measured as described in Nebauer et al. (2011). Corrections for leakage of CO_2_ into and out of the leaf chamber of the LI-6400 were applied to all the gas exchange data (Flexas et al., 2007). From the combined gas exchange and chlorophyll a fluorescence measurements, mesophyll conductance to CO_2_ (g_m_) was estimated according to Harley et al. (1992). Six independent curves were produced per treatment and genotype.

### RNA isolation and RT-qPCR analyses

Total RNA was extracted using RNeasy Plant Mini Kit (Qiagen). The gene expression levels in the transgenic tomato plants were determined by RT-qPCR following the procedures described in Corrales et al. (2014). The primer pairs used for amplification are described in Table S1. The *UBIQUITIN3* gene from *S. lycopersicum* was used as the reference gene (Hoffman et al., 1991). The relative expression levels of the target genes were calculated by the 2^−ΔΔCT^ method (Livak and Schmittgen, 2001). Presence of Dof binding sites in the promoters (1,500 pb) of the selected genes was established with the PlantPan2 software (Chow et al., 2016).

### Transcriptomic analysis

Differences in gene expression were studied by RNA-Seq in the 45-day-old NT and *35S::AtCDF3* (L 2.3) plants grown under the control and salinity conditions (75 mM NaCl) using an Illumina HiSeq2000 platform (BGI, China). Total RNA was extracted from leaves and purified with the Qiagen RNeasy extraction kit according to the manufacturer's instructions, and was treated with RNase-free DNase (Ambion). RNA integrity and quantity were confirmed with the Bioanalyzer 2100 (Agilent). cDNA synthesis, adaptor ligation and sequencing were performed by BGI. The raw Illumina sequencing data are deposited in GEO (http://www.ncbi.nlm.nih.gov/geo/) at NCBI.

The *S. lycopersicum* genome and gene information were downloaded from Solgenomics (ftp://ftp.solgenomics.net/genomes/Solanum_lycopersicum/annotation/ITAG2.4_release/ITAG2.4 Release genomic annotation). After removing the reads that contained sequencing adapters and reads of low quality (reads that contained Ns >5), the remaining reads were aligned to the *S. lycopersicum* genome using SOAP (Li et al., 2009). The reads that failed to be mapped and the paired-end reads were processed as described by Wang et al. (2010).

The gene expression level by RNA-Seq was normalized by the number of reads per kilobase of exon region per million mapped reads (RPKM) (Mortazavi et al., 2008). The cut-off value for the determining gene transcriptional activity was determined based on a 95% confidence interval for all the RPKM values of each gene. The transcript fold change was calculated as the log_2_ of the ratio between two samples. Here, genes with a fold change >2.0 were considered to be differentially expressed.

The common genes that are represented in one or more data sets were identified using the web-based VENNY tool (http://bioinfogp.cnb.csic.es/tools/venny/) Finally, Gene Ontology analyses were performed using the agriGO (http://bioinfo.cau.edu.cn/agriGO/; Du et al., 2010) and REVIGO (http://revigo.irb.hr/; Supek et al., 2011) software.

### Metabolomic analyses

The total soluble sugars and starch content in leaves and roots were determined colorimetrically by the anthrone method, as described in Nebauer et al. (2011). The NT and *35S::AtCDF3* (L 2.3 and 10.1) plants were grown under the control and salinity conditions for 15 days. Three independent leaf extracts were used for the analyses in any treatment and genotype. Targeted metabolomics analyses were also performed. Extraction, manipulation and mass spectrometric analyses of samples followed an adapted protocol described in Corrales et al. (2014).

### Determination of mineral elements content

Sodium, calcium, magnesium, potassium, and phosphate contents were determined in the leaves, stems and roots of 45-day-old NT and *35S::AtCDF3* (L 2.3) plants grown under control and salinity (75 mM NaCl) conditions for 15 days with an ICP-AES (Thermo Scientific, USA). For each treatment and genotype, plant material was dried for 48 h at 60°C and finely ground in a Pulverisette mill (Fristch, Germany). Acid digestion of samples was done by the method explained in Nebauer et al. (2011).

### Hormone determinations

Hormones (jasmonic acid, indole-3-acetic acid, isopentenyl adenine, abscisic acid and GA_12_, GA_15_, GA_24_, GA_9_, GA_51_, GA_4_, GA_34_, GA_53_, GA_44_, GA_19_, GA_20_, GA_29_, GA_1_, and GA_8_ gibberellins) were analyzed in leaves of NT and *35S::AtCDF3* (L 2.3) plants grown under control and salinity conditions for 15 days by liquid chromatography-electrospray ionization-tandem mass spectrometry (LC-ESI-MS/MS) using a Q-Exactive spectrometer (Orbitrap detector; ThermoFisher Scientific) by the Plant Hormone Quantification Service, IBMCP, Valencia, Spain.

### Determination of tomato production and fruit quality

The agronomic performance of the transgenic lines was assessed in a third experiment by measuring total yield (g plant^−1^), number of fruits and fruit weight (g fruit^−1^). All fruits were harvested at maturity in each plant until the 6th truss. Plants were obtained as described above (Renau-Morata et al., 2014) and seedlings were transferred to 15 L pots that contained coconut fiber, and irrigated with half-strength Hoagland no. 2 nutrient solution (Hoagland and Arnon, 1950). Plants were cultured in the greenhouse from December to mid-August. Salinity was imposed by adding 75 mM of NaCl to the irrigation solution. Plants were irrigated twice a week with excess solution to ensure optimal watering and to minimize salt accumulation in the substrate during the experiment. No significant incidence of Blossom-End Rot (BER) was observed among the studied lines.

Four representative fruits were collected from each plant in the mature-red stage (only from the first three trusses to minimize intraplant variability). Soluble solids content was measured by refractometry. Taste components were determined by capillary electrophoresis, as described by Cebolla-Cornejo et al. (2012). Sugars fructose, glucose and sucrose, and organic acids malic, citric, glutamic acid and γ-amino butyric acid (GABA), were quantified. The derived sucrose equivalents, sucrose equivalents/citric acid and sucrose equivalents/malic acid ratios were calculated. Carotenoids β-carotene and lycopene were determined by reversed phase HPLC, as described in Cortés-Olmos et al. (2014).

### Statistical analyses

Data were analyzed by a two-way ANOVA (genotype and treatment) or one-way ANOVA with the Statgraphics statistical software (Statgraphics Centurion XVI, Statpoint Tech, Inc., USA). The treatment mean values were compared (*P* < 0.05) by Fisher's least significant difference (LSD) procedure. A regression analysis (*P* < 0.05) was used to evaluate the relationships between parameters.

## Results

### Generation of tomato plants that overexpress *AtCDF3* and *SlCDF3* genes

We have previously identified a group of tomato DOF TFs (*SlCDFs*) that exhibit specific expression patterns in response to diverse environmental stresses and whose functions are related to abiotic stress tolerance and flowering time (Corrales et al., 2014). Among them, *SlCDF3* was particularly interesting for its high induction under precise stress conditions. Similar observations have been recently reported for the putative orthologous Arabidopsis *AtCDF3* gene (Corrales et al., 2017), assigning an important role to this TF in regulating abiotic stress responses in plants.

To further study the function of CDF3 and to determine its impact on plant growth and production, a phenotypic characterization of *CDF3* gain-of-function plants was accomplished by analyzing their performance under control and abiotic stress conditions (e.g., salinity). Tomato plants (cv. Moneymaker) overexpressing *AtCDF3* or *SlCDF3* genes were obtained after transformation with a binary vector harboring the corresponding coding sequences under control of the constitutive CaMV 35S promoter.

**Figure 1:**
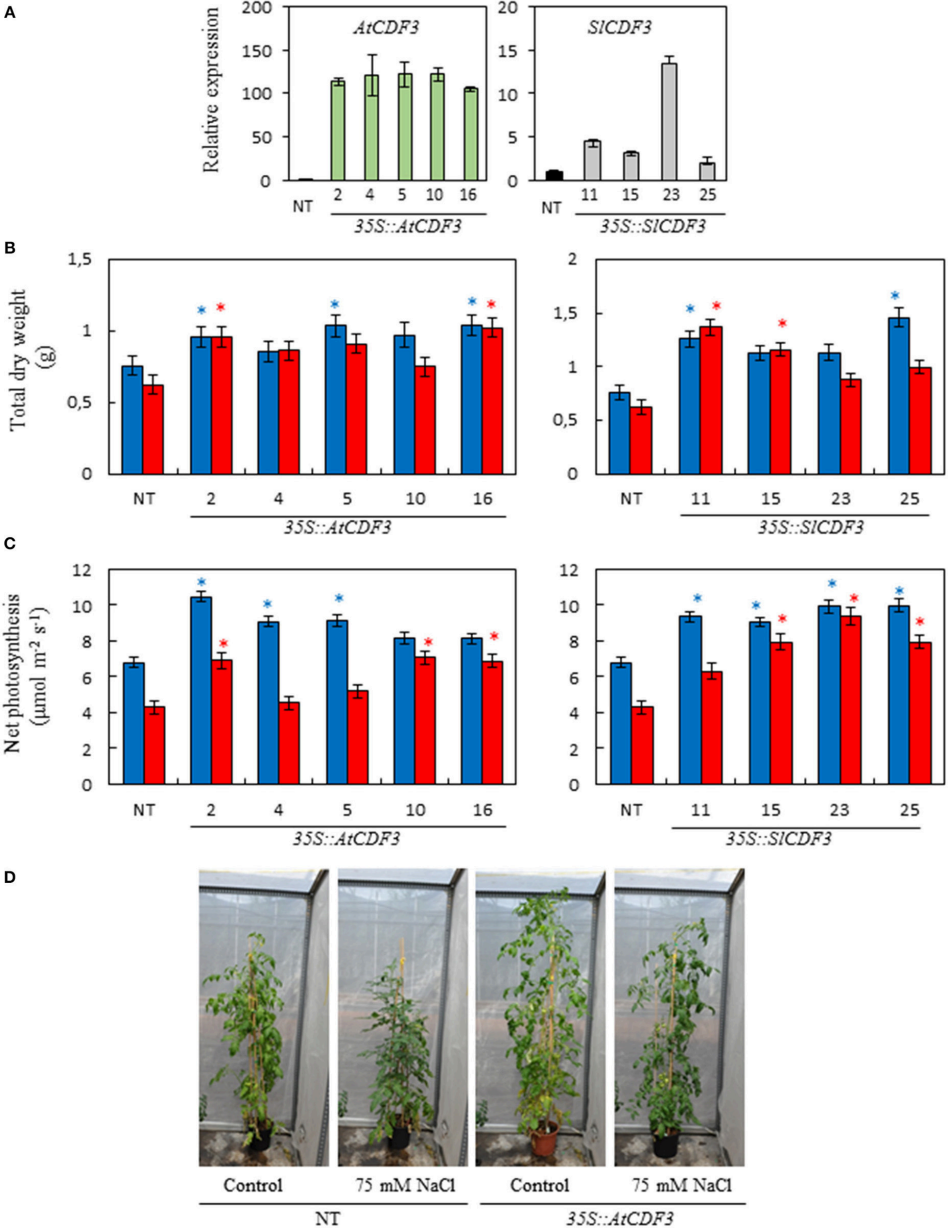
**Phenotypic characterization of the ***35S::AtCDF3*** and ***35S::SlCDF3*** tomato lines. (A)** Expression levels of the corresponding gene *AtCDF3* or *SlICDF3* analyzed by RT-qPCR. **(B)** Biomass and **(C)** photosynthetic rate of the *35S::AtCDF3, 35S::SlCDF3* and NT tomato lines grown under the control (blue bars) and 75 mM NaCl (red bars) conditions. Thirty-day-old plants grown in hydroponic culture were subjected to moderate salinity (75 mM NaCl). Photosynthesis and growth determinations were performed after 15 days of treatment. **(D)** Representative examples of the NT and *35S::AtCDF3* (line 2.3) plants grown under the control or salt stress conditions in the greenhouse. Asterisks indicate significant differences (*P* < 0.05) of the transgenic plants with the non-transformed plants (NT) under the same growth condition. NT, non-transformed.

From each transformation, individual lines were selected (L 2, 4, 5, 10, and 16 of *35S::AtCDF3* plants; and L 11, 15, 23, and 25 of *35S::SlCDF3* plants). These lines were analyzed by RT-qPCR to test the expression levels of the inserted *CDF3* genes (Figure 1A). The transcript levels of both genes were significantly higher in the tested transgenic lines compared to NT (non-transformed) plants.

### Overexpression of *AtCDF3* and *SlCDF3* improves biomass production under control and salinity stress conditions

A phenotypic characterization of the *CDF3* overexpressing plants was conducted by analyzing their responses under control and salinity conditions. Plant biomass was determined in the *35S::AtCDF3* (L 2, 4, 5, 10, and 16) and *35S::SlCDF3* (L 11, 15, 23, and 25) and NT plants that were either grown under control conditions or treated with 75 mM NaCl. *35S::CDF3* and NT tomato plants grown under control conditions in hydroponic cultures did not show apparent developmental differences. However, *CDF3* overexpressing plants consistently exhibited a larger size than the NT plants (Figure 1B). Accordingly, the majority of the *35S::CDF3* plants displayed significantly higher dry weight values than the NT plants under these conditions. This higher total dry weight was due to the increased size of all the plant organs (*r* > 0.90^**^, data not shown).

*35S::AtCDF3* and *35S::SlCDF3* plants also displayed increased biomass under the salinity conditions (Figures 1B,D). Although salinity reduced plant growth in some lines under the assayed conditions, the plants overexpressing *CDF3* genes consistently exhibited higher total biomass than the NT plants.

### Enhanced photosynthetic capacity of *AtCDF3* and *SlCDF3* overexpressing tomato plants

To investigate the underlying mechanisms involved in the observed growth responses of *35S::AtCDF3* and *35S::SlCDF3* plants, we examined different physiological parameters such as net photosynthesis and related gas exchange variables, stomatal conductance and substomatal CO_2_ concentration using a LI-6400 infrared gas analyser. NT and *35S::CDF3* plants were grown in hydroponic culture for 30 days, exposed to salinity (75 mM NaCl) for 15 days, and the photosynthetic parameters were measured.

As shown in Figure 1C, under the control conditions most of the 35S::*AtCDF3* and 35S::*SlCDF3* plants displayed higher photosynthetic rates than the NT plants. Although salt stress conditions reduced the photosynthetic rate values, most of the *AtCDF3* and *SlCDF3* overexpressing lines also exhibited higher CO_2_ fixation rate values than the NT plants in these conditions (Figure 1C). Both the Arabidopsis and tomato *CDF3* genes consistently promoted a similar effect (*P* < 0.05).

The high correlation between the photosynthetic rate and total plant biomass observed (Total dry weight = 0.075^*^A_N_+ 0.40; *r* = 0.85; *P* = 0.00), indicates that increased photosynthesis sustains the higher growth rate observed in the plants overexpressing *CDF3* genes.

We further investigated diverse photosynthetic parameters (Table 1) from combined gas exchange and chlorophyll fluorescence analyses in the *35S::AtCDF3* plants (L 2.3). The higher photosynthetic rate in the plants that overexpress *AtCDF3*, compared to the NT plants under the control conditions (10.8 vs. 8.5 μmol m^−2^ s^−1^) could be related to higher stomatal conductance (Table 1), suggesting lower limitations to CO_2_ diffusion to the mesophyll in transgenic plants. The *35S::AtCDF3* plants exhibited a higher effective quantum yield of PSII (PhiPS2) indicating a higher proportion of absorbed light used in photochemistry. Both the higher performance of the electron transport chain in the thylakoid membranes, and the higher maximum carboxylation rate of Rubisco (V_c max_) in the CO_2_ fixation reactions (Table 1), support an improved overall photosynthesis.

**Table 1:** **Effect of salinity on photosynthetic parameters in Moneymaker tomato (NT) and ***35S::AtCDF3*** plants**.

		**A**_**N**_			**g**_**s**_			**g**_**m**_			**C**_**i**_			**PR**			**PhiPS2**			**F**_**v**_**/F**_**m**_		**V**_**cmax**_			**J**_**max**_			**TPU**		
		**μmol m**^**−2**^ **s**^**−1**^			**mol m**^**−2**^ **s**^**−1**^			**mol m**^**−2**^ **s**^**−1**^			**μmol mol**^**−1**^			**μmol m**^**−2**^ **s**^**−1**^								**μmol m**^**−2**^ **s**^**−1**^			**μmol m**^**−2**^ **s**^**−1**^			**μmol m**^**−2**^ **s**^**−1**^		
NT	Control	8.5	a		0.41	a		0.12			353	a		2.4			0.09			0.83		93			88			5.4	a	
	Salinity	6.1	b		0.20	b		0.13	ns		326	b		2.7	ns		0.09	ns		0.83	ns	86	ns		87	ns		4.4	b	
*35S::AtCDF3*	Control	10.8	a	^*^	0.61	a	^*^	0.14	a		354	a		2.6	b		0.12		^*^	0.83		111		^*^	92			5.3	b	
	Salinity	9.4	b	^*^	0.41	b	^*^	0.17	b	^*^	346	b	^*^	3.5	a	^*^	0.12	ns	^*^	0.84	ns	102	ns	^*^	103	ns	^*^	5.9	a	^*^

*Thirty-day-old plants grown in hydroponic culture were subjected to moderate salinity (75 mM NaCl). Photosynthesis determinations were performed after 24 days. Photosynthetic rate (A_N_), stomatal conductance (g_s_), mesophyll conductance (g_m_), substomatal CO_2_ concentration (C_i_), photorespiration rate (PR), effective (PhiPS2) and maximum (F_v_/F_m_) quantum yield of chlorophyll fluorescence of PSII, maximum carboxylation rate of Rubisco (V_c max_), maximum electron rate (J_max_) and triose phosphate utilization rate (TPU) values in 35S::AtCDF3 (L 2.3) plants are presented. Each measure is the mean of ten independent measurements in different plants. Six different plants were used to perform A-Cc curves to obtain V_c max_, J_max_ and TPU*.

*For each genotype and parameter, different letters indicate significant differences (P < 0.05). Significant differences among genotypes at a given condition are indicated by an asterisk*.

Salinity caused the photosynthetic rate to drop in both the NT and *35S::AtCDF3* (L 2.3) plants (Table 1). However, this reduction was less marked in the transgenic plants (11 vs. 28% in the NT plants). Although stomatal closure was also provoked by salinity, stomatal conductance in the transgenic plants was also higher than in the NT plants, which allowed better CO_2_ diffusion into leaves. The greater mesophyll conductance observed in the *35S::AtCDF3* plants also improved the CO_2_ supply from substomatal air spaces to the stroma in the chloroplast. Under salinity, the transgenic plants also exhibited the higher effective quantum yield of PSII and the maximum carboxylation rates of Rubisco reported under the control conditions. Interestingly, the plants that overexpress the *AtCDF3* gene showed higher triose phosphate utilization rates under these stress conditions (Table 1), which suggests that the carbon flux to sucrose and starch synthesis is maintained in these plants.

Taken together, our data indicate that the greater biomass accumulation observed in the plants that overexpress the *AtCDF3* and *SlCDF3* genes is sustained by a higher photosynthetic rate, and that the greater photosynthetic capacity of these plants could be related to both improved diffusional and biochemical factors.

### Transcriptome analysis of transgenic tomato plants overexpressing CDF3 genes

**Figure 2:**
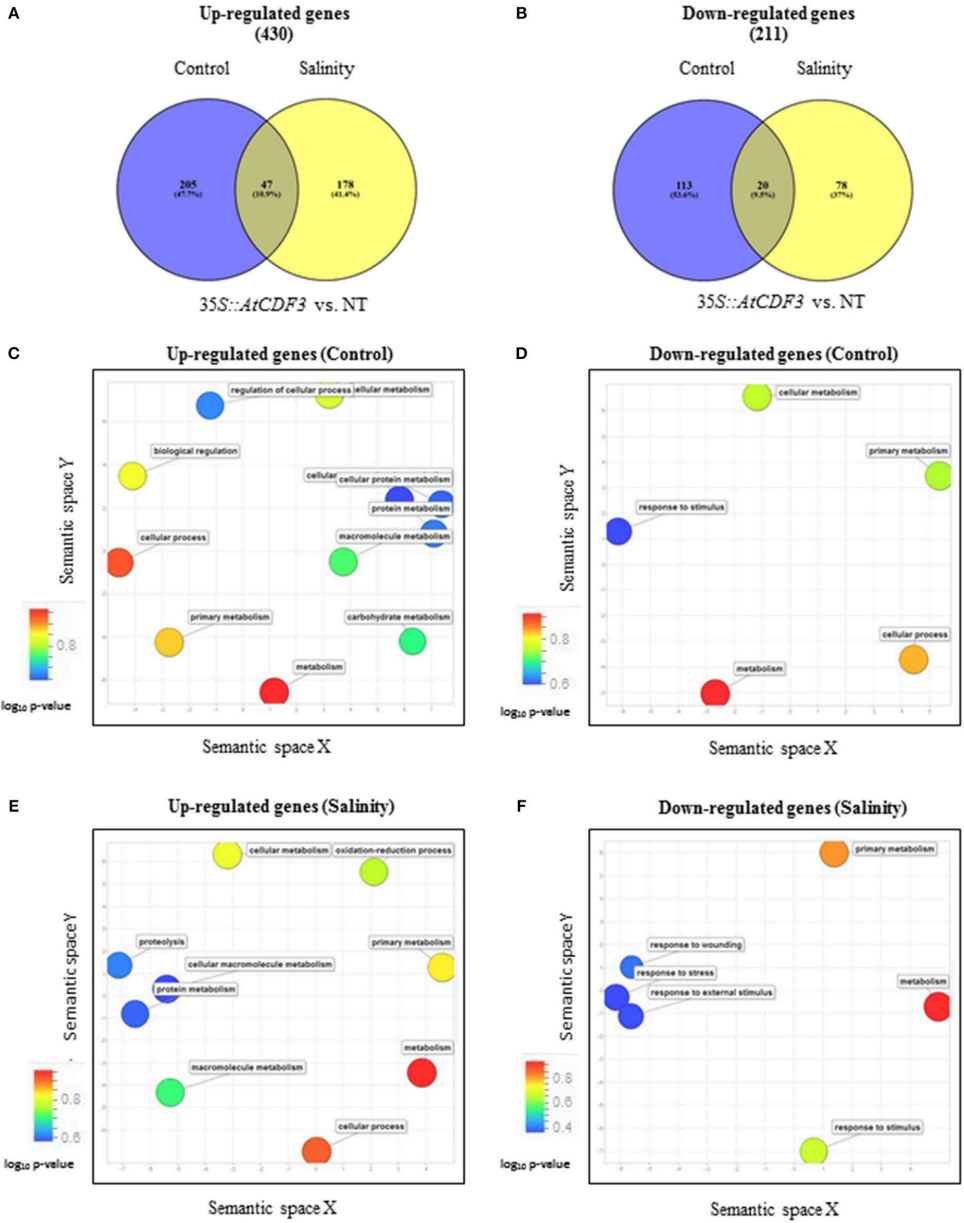
**Classification and gene ontology analyses of the genes differentially expressed in the ***35S::AtCDF3*** plants compared to the NT plants under control and salinity conditions**. Thirty-day-old NT and *35S::AtCDF3* plants grown in hydroponic culture were subjected to moderate salinity (75 mM NaCI). Leaf transcriptomic analysis was performed after 15 days. **(A,B)** Venn diagrams showing the overlap of the up-regulated and down-regulated genes expressed in the *35S::CDF3* transgenic plants vs. the NT plants in response to salinity. **(C–F)** Scatter plot of the genes significantly (*P* < 0.05) up- or down-regulated in the *35S::AtCDF3* plants compared with the non-transformed NT plants under control and salinity conditions. The GO analysis was performed using the AgriGO and Revigo tools. Bubble color indicates the *P*-value for the false discovery rates derived from the AgriGO analysis as well as biological processes. NT, non-transformed.

Transcriptome analysis of 45-day-old NT and *35S::AtCDF3* (L 2.3) plants grown under control and salinity conditions was performed by RNA-seq. Leaf transcriptomes of *35S::AtCDF3* and NT plants grown under control conditions revealed 385 differentially expressed genes (DEGs: >2-fold change, *P* < 0.05, see Figures 2A,B). Of them, 252 were up-regulated and 133 down-regulated (Table 2; Tables S2, S3). Furthermore, GO (gene ontology) analyses revealed that CDF3-regulated genes are highly enriched in terms related to the primary metabolism and regulation processes (Figures 2C,D). Among the up-regulated genes in the transgenic plants and directly linked to the phenotypes observed are the genes involved in carbon and nitrogen metabolism, like phosphoenolpyruvate carboxykinase (PEPCK, Solyc04g076880.2.1) and pyruvate kinase (PK, Solyc03g007810.2.1) participating in glycolysis and gluconeogenesis; and glutamate synthase (GS, Solyc01g080280.2) and glutamate decarboxylase (GAD, Solyc11g011920.1) involved in nitrogen assimilation. Likewise, a set of genes that improve photosynthetic efficiency and redox balance, including DNAJ chaperones (*DnaJ8*, Solyc11g011920.1, *DnaJ11*, Solyc07g065970.1.1, and *DnaJ20*, Solyc09g092260.2.1), TRX thioredoxin (*Cxx1*, Solyc02g079960.2.1) and peroxidases (Solyc03g119060.2.1, Solyc02g092580.2.1 and Solyc04g071890.2.1), are also up-regulated in the *35S::AtCDF3* plants (Table 2).

**Table 2:** **Summary of representative up-regulated genes in the transgenic ***35S::AtCDF3*** (L 2.3) tomato plants under control and salinity conditions**.

	**Processes/activities**	**Genes**
Control	Transcription factors	NAC, WRKY, TCP, AP2, PIF, MYB, HS, MADS-box -type
	Hormone responses	Related to ABA, auxins, gibberellins, strigolactones
	C and N metabolism	Asparagine synthase, glutamate synthase, PEP carboxykinase, pyruvate kinase, glutamate decarboxylase
	Transporters	Aquaporins, nitrate, amino acid, ATP/ADP
	Cell growth	Expansin, cellulose synthase, XTH
	Protective roles	DnaJ, thioredoxins, catalase, peroxidase
Salinity	Transporters	Phosphate, nitrate, amino acid, sugars, calcium, copper
	Hormone responses	Related to ethylene, auxins, cytokinins
	Transcription factors	NAC, WRKY, PIF, TCP, HS -type
	Photosynthesis	PSII related, oxidoreductases, ATP synthase
	Cell growth	Cellulose synthase, expansin

*Transcriptomic analysis by RNAseq was performed in 30d-old plants subjected to salinity (75 mM NaCl) for 15 days*.

Other important genes related to plant growth are induced in the *35S::AtCDF3* plants. Among them, trehalose-6-phosphate (T6P) synthase (Solyc07g006500.2.1), which is involved in the synthesis of the sucrose sensor molecule T6P that modulates growth and stress responses (Ruan, 2014), or genes that participate in cell wall expansion, like expansins (Solyc08g077900.2.1 and Solyc08g077330.2.1), xyloglucan endo transglycosylase/hydrolase (*XTH*, Solyc05g046290.2.1) and cellulose synthase (*CESA*, Solyc03g005450.2.1). Among the up-regulated transcription factors, IAA29 (Solyc08g021820.2.1) and PIF1 (Solyc06g008030.2.1) have been reported to participate in the control of plant growth (Leivar and Monte, 2014; Huang et al., 2015; Shimizu et al., 2016), and other TFs like NAC29, MYB44 (Solyc04g078420.1.1) and WRKY33 (Solyc06g066370.2.1) involved in ABA and stress responses. Altogether, these data suggest that CDF3 might function in the regulation of plant growth through processes related to photosynthesis, C/N metabolism and cell growth under variable environmental conditions.

A similar analysis of plants grown under the salinity conditions, also revealed differentially expressed genes between the NT and *35S::AtCDF3* plants (225 up-regulated and 98 down-regulated genes, see Figures 2A,B). Primary metabolism, transport and regulation processes were over-represented terms in the GO analyses of DEGs (Figures 2E,F). Notably, several transporter activities, TFs related to growth and stress responses like WRKY40 (Solyc08g067340.2.1), PIF1, NAC22 (Solyc07g066330.2.1) and TCP12 (Solyc11g020670.1.1), and genes related to photosynthesis performance, including thylakoid proteins or redox components, were also up-regulated in the *35S::AtCDF3* plants (Table 2; Tables S4, S5).

**Figure 3:**
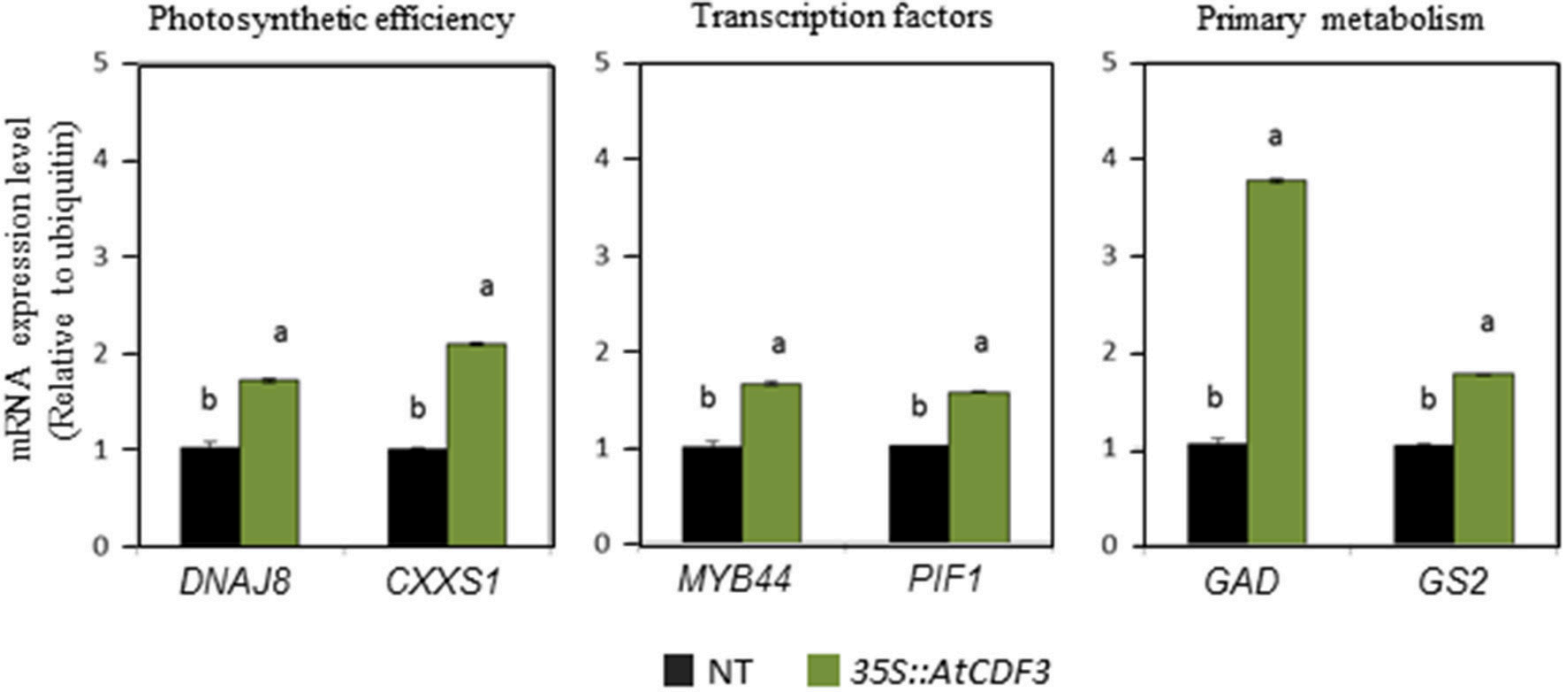
**AtCDF3 regulates the expression of genes involved in photosynthesis, primary metabolism and growth-and stress- related transcription factors**. Transcription analysis by RT-qPCR of genes *DNAJ8, CxxSl, MYB44, PIFl, GAD*, and *GS2* in the leaves of the *35S::AtCDF3* (green bars) and NT (black bars) tomato plants grown under the control conditions for 30 days. *UBIQUITIN3* gene expression was used as the reference gene. Data are expressed as mean ± SE of three independent extractions. Three technical replicates were performed for each extraction. For each gene, different letters indicate significant differences (*P* < 0.05).

Quantitative RT-PCR was performed to confirm some of the identified differentially expressed genes in the tomato *35S::AtCDF3* plants. This analysis included the genes that codify for the TFs involved in the regulation of growth and stress responses, like *PIF1* and *MYB44*, genes related to C/N metabolism, like glutamate synthase *GS2* and glutamate decarboxylase *GAD*, and genes related to the protection of the photosynthetic process, like *CxxS1* thiorredoxin and the *DnaJ8* chaperone. Figure 3 shows the expression levels of the analyzed genes in the *35S::AtCDF3* transgenic plants, where they exhibit higher values (from 2- to 4-fold) than in the NT plants. These data confirmed the results of the RNA-seq analyses and points to CDF3 as a potential upstream activator in photosynthesis, growth and stress pathways. Interestingly, several DOF binding sites were found in the promoters of the analyzed genes in Figure 3 using the PlantPan2 software (data not shown), suggesting that they might be direct target genes of the CDF3 transcription factor.

### Overexpression of *AtCDF3* in vegetative tissues impacts sugar and amino acid metabolism in tomato

**Figure 4:**
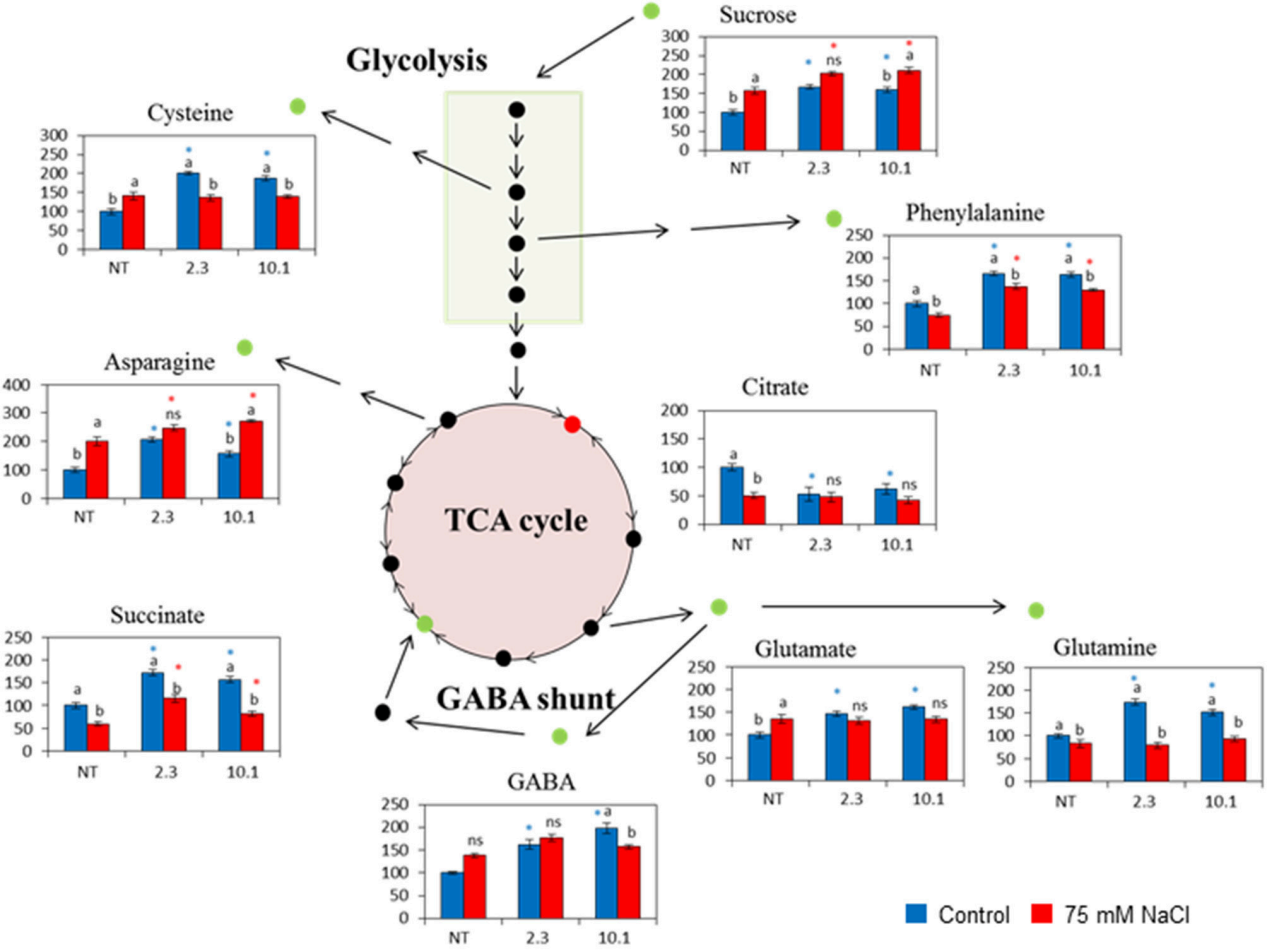
**Schematic representation of the altered metabolism in the ***35S::AtCDF3*** plants (lines 2.3 and 10.1) under the control (blue bars) and salinity (red bars) conditions**. Thirty-day-old NT and *35S::AICDF3* plants (L 2.3 and 10.1) were grown in hydroponic culture under control and salinity (75 mM NaCl) conditions. Metabolomic determinations in leaves were performed after 15 days. The relative quantities (% of NT) of the selected metabolites analyzed by gas chromatography-selected ion monitoring-mass spectrometry are displayed. Different letters indicate the significant differences (*P* < 0.05) within each genotype for the stress effect. Differences between genotypes per treatment are indicated by an asterisk. NT, non-transformed.

We performed a targeted metabolomic profiling by gas chromatography-mass spectrometry (GC-MS) to study the relative levels of the different polar compounds, including proteinogenic amino acids, and other amino acids and distinct sugars, extracted from the 45-day-old NT and two *35S::AtCDF3* tomato lines (L 2.3 and 10.1) either grown under the control conditions or treated with 75 mM of NaCl for 15 days (Figure 4; Figure S2).

Figure 4 shows metabolite levels under control conditions, whereby the overexpression of *AtCDF3* induced the accumulation of sugars like sucrose, organic acids like succinate and amino acids like glutamate, glutamine, asparagine, phenylalanine, cysteine and GABA in both lines, while the amounts of tryptophan, citrate and gluconate decreased in comparison to the NT plants. No changes in leaf starch were observed (data not shown) and glucose and fructose amounts remained unchanged compared to NT plants (Figure S2). Overall, the increased photosynthetic rate in the *35S::AtCDF3* plants favors the production of sucrose available for transport and growth, and increased the content of amino acids glutamate and glutamine, related to N assimilation.

Under the salt stress conditions, a reduction in fructose, citrate, tryptophan and aspartate was observed, but only in the NT plants (Figure 4; Figure S2). Succinate, phenylalanine and glutamine levels lowered in all genotypes. Sucrose and glutamate accumulated under salinity in the NT plants at similar levels to those observed in the overexpressing plants. Interestingly, under these lower C availability conditions, N assimilation was channeled in the form of asparagine in the *35S::AtCDF3* plants.

### The overexpression of *CDF3* genes in tomato increases tomato yield under the control and saline conditions and improves fruit quality traits

Since the *AtCDF3* and *SlCDF3* overexpression in tomato promoted higher photosynthesis and biomass production, we investigated the impact of these TFs on plant yield. To this end, greenhouse assays were performed to characterize the agronomic performance of the *35S::AtCDF3* (L 2.3 and 10.1), *35S::SlCDF3* (L 11.2 and 23.1) and NT plants. Total yield, number of fruits and fruit weight were measured in the selected lines, both under control and saline conditions (75 mM of NaCl).

**Figure 5:**
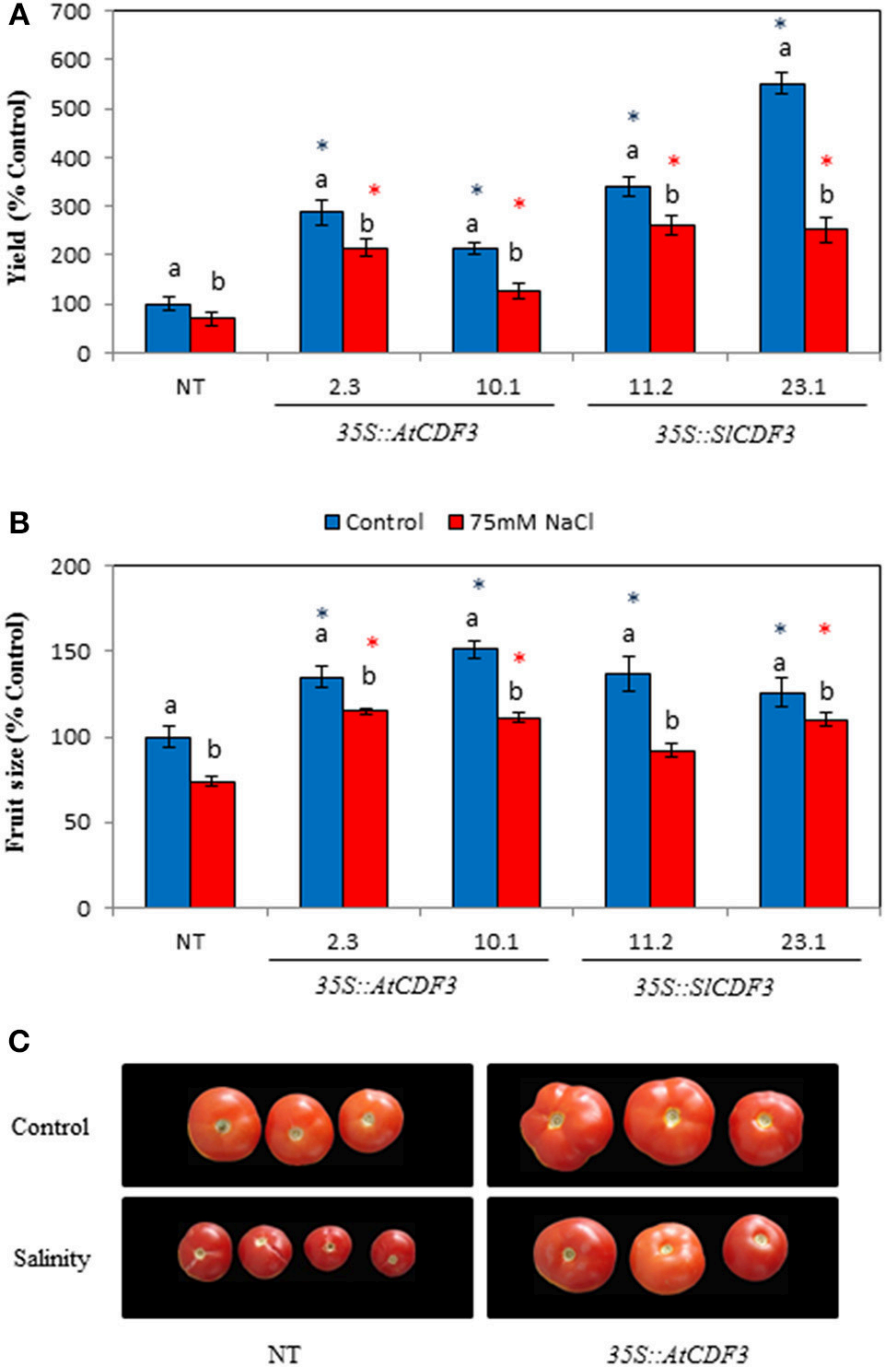
**Agronomic performance of tomato plants overexpressing ***AtCDF3*** or ***SlCDF3*** genes under control and salinity conditions. (A)** Yield and **(B)** fruit size of tomato plants under control (blue bars) and salinity (75 mM NaCl; red bars) conditions. Plants were cultured in the greenhouse from December to mid-August. All fruits were harvested at maturity in each plant until the 6th truss. The results are provided as a percentage of yield or fruit size in relation to the NT plants grown under the control conditions. **(C)** Representative examples of the tomatoes from the NT or *35S::AtCDF3* (line 2.3) plants grown under control or salt stress. Asterisks indicate significant differences (*P* < 0.05) with the NT plants grown under the same condition. Letters indicate significant differences for a given genotype between the control and salinity conditions. NT, non-transformed.

The results in Figure 5A show that under the non-stress control conditions both the *35S::AtCDF3* and *35::SlCDF3* plants present improved fruit yield (up to 550% of yield in NT plants). Notably, the production was related to the photosynthetic rate (Total yield = 36.1^*^A_N_-68.4; *r* = 0.74; *P* = 0.02) and the improved yield in the tomato transgenic plants resulted mainly from a larger number of fruits than the NT plants. In addition, the fruit size was larger (up to 137%) in the plants overexpressing CDF3 than in the NT plants (Figures 5B,C). However, increased fruit size influenced the agronomic performance of transgenic plants, but to a lesser extent than number of fruits.

Under salt stress conditions, tomato yield decreased in almost all the lines, but was always higher in the *35S::CDF3* plants, which achieved up to 250–280% of NT plants. These results were confirmed in a second experiment performed with two additional *35S::AtCDF3* lines (L 2.3 and 10.1) in a different year (data not shown). As expected, tomato fruit size was reduced when the transgenic plants were exposed to salt stress. Under these conditions, the fruit size of the overexpressing lines was, nevertheless, greater than that of the NT plants.

**Figure 6:**
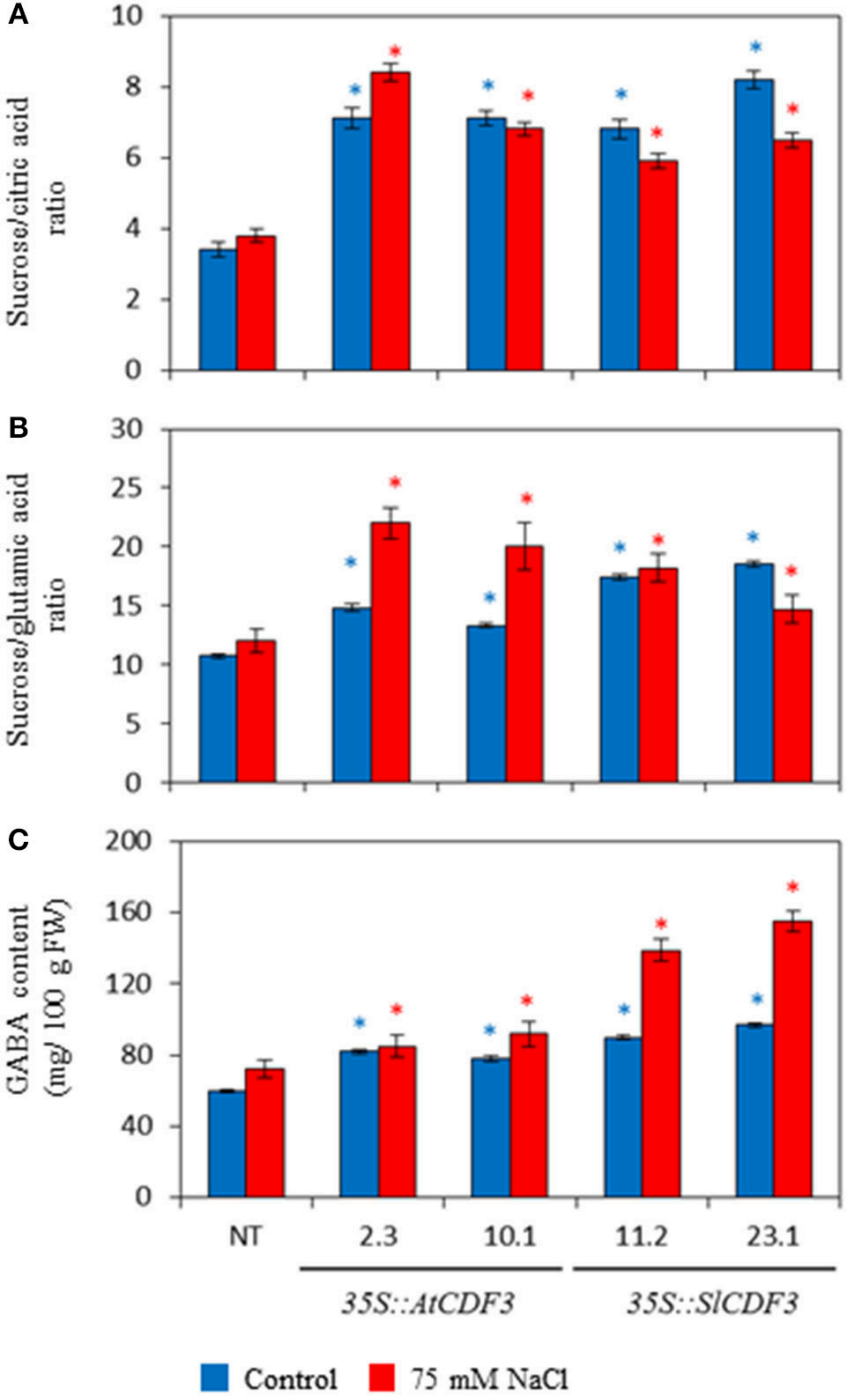
**Effect of the overexpression of the Arabidopsis and tomato ***CDF3*** genes on the compounds and variables related to organoleptic quality in tomato fruits**. *35S::AtCDF3* (lines 2.3 and 10.1) or *35S:SlCDF3* (lines 11.2 and 23.1) and NT tomato plants were cultured under control (blue bars) and salinity (75 mM NaCl, red bars) in the greenhouse from December to mid-August. Four representative fruits, until the 3rd truss, were collected from each plant in the mature-red stage. The sucrose equivalents/citric acid **(A)** and sucrose equivalents/glutamic acid **(B)** ratios and GABA content **(C)** are displayed. Asterisks indicate significant differences (*P* < 0.05) of the transgenic plants with the non-transformed plants (NT) under the same growth condition.

We further analyzed metabolites and variables related to organoleptic quality in the fruits of the *35S:*:*AtCDF3* (L 2.3 and 10.1) and *35S::SlCDF3* (L 11.2 and 23.1) plants under control and salinity conditions. Remarkably, overexpression of *CDF3* genes had a strong impact on organic acid and sugar accumulation, and in the derived variables related to flavor perception. Figure 6 and Table S6 display important changes in the transgenic lines compared to the NT plants, as a higher malic to citric acid ratio or higher GABA and glucose content (e.g., 35% higher glucose content in the *35S::SlCDF3* line 23; Table S6). In general, overexpression of the *CDF3* genes led to higher sucrose equivalents to the citric and glutamic acid ratios, parameters that strongly correlate with sensorial perception.

Under salinity stress, dry matter, soluble solids and glucose content of fruits of the NT and *CDF3* overexpressing plants moderately increased. Likewise, organic acids and fructose also increased (Figure 6; Table S6), but the reported lower citric acid and increased malic acid contents were maintained in the fruits of the transgenic plants. These plants also presented higher fructose and glucose levels, as well as higher sucrose and sucrose equivalents to the citric and glutamic acid ratios, as observed in the control media.

Overall, these data indicate that overexpression of the *CDF3* genes in tomato improves yield due to increased fruit number and weight, and also improves quality under control conditions. As expected, salinity reduces yield in both the NT and most *35S::CDF3* plants, although fruit production always remained higher in the transgenic lines.

### Effects of *AtCDF3* overexpression on ion accumulation

**Figure 7:**
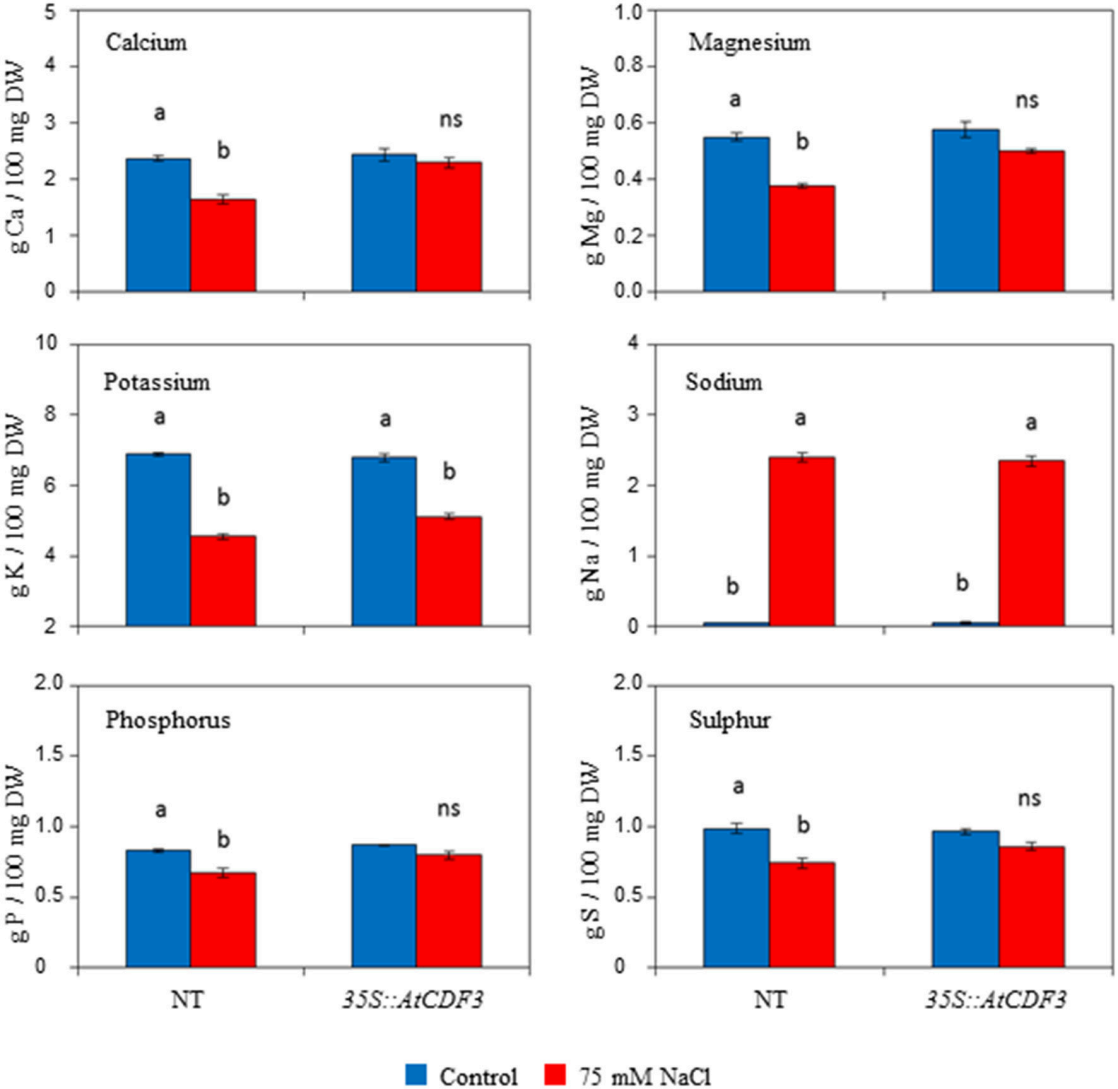
**Effect of ***AtCDF3*** overexpression on leaf mineral elements content in tomato**. Thirty-day-old plants grown in hydroponic culture were subjected to moderate salinity (75 mM NaCl). Mineral elements determinations were performed after 15 days of treatment. The calcium, magnesium, sodium, potassium, phosphor, and sulfur levels were shown under the control (blue bars) and salinity (red bars) conditions in the NT and *35S::AtCDF3* plants (line 2.3). Different letters indicate the significant differences (*P* < 0.05) within each genotype for the stress effect. NT, non-transformed.

Since salinity reduces nutrient uptake and affects nutrient partitioning within plants (Grattan and Grieve, 1999), we wanted to investigate whether the improved yield of the *35S::CDF3* plants under salt stress was due to reduced sodium accumulation and lower toxicity, and test the mineral composition in the transgenic plants. Growth inhibition is often correlated with a high internal Na^+^ and K^+^ deficiency in vegetative tissues. Total content of sodium, calcium, magnesium, potassium and phosphate was determined in leaves, stems and roots of the 45-day-old *35S::AtCDF3* (L 2.3) and NT plants grown under control and salinity conditions for 15 days (Table S7). Notably, no significant differences were observed between the transgenic and NT plants under the control conditions. Under salinity both genotypes accumulated similar levels of sodium in the different analyzed organs, and although some reduction in the amount of potassium was triggered, no differences were observed between the NT and transgenic plants (Figure 7; Table S7). It should be noted that salinity provoked smaller leaf amounts of essential elements magnesium, calcium, phosphor and sulfur in the NT plants (Table S7). In contrast, the content of these elements were maintained in the *35S::AtCDF3* plants, which thus avoided mineral deficiency triggered by salt stress.

### Tomato plants overexpressing *AtCDF3* show increased content of active gibberellins

**Figure 8:**
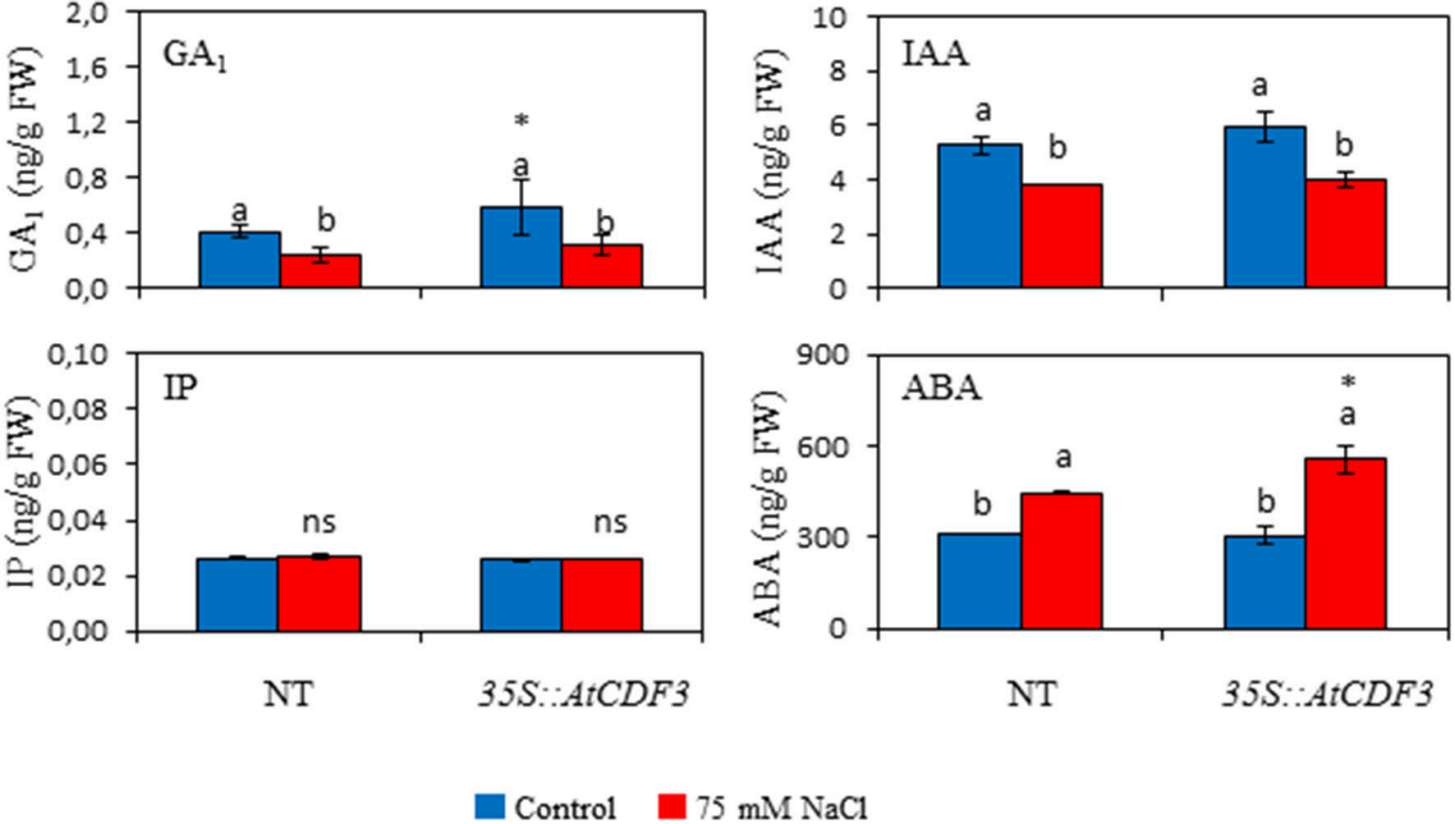
**Effect of ***AtCDF3*** overexpression on leaf hormone content in tomato**. Thirty-day-old NT and *35S::AtCDF3* (L 2.3) plants grown in hydroponic culture were subjected to control and moderate salinity (75 mM NaCl) conditions. Hormone determinations were performed after 15 days of treatment. The GA_1_, IAA, IP, and ABA levels in leaves were shown under the control (blue bars) and salinity (red bars) conditions. Different letters indicate the significant differences (*P* < 0.05) within each genotype for the stress effect. Differences between genotypes per treatment are indicated by an asterisk. NT, non-transformed.

The role of hormones in the regulation of plant growth and development and in responses to stresses is well-known (Albacete et al., 2014). To investigate whether the improved growth and yield observed in the *35S::CDF3* plants could be related to changes in hormone contents, gibberellins (GAs), jasmonic acid (JA), isopentenyladenine (IP), indole acetic acid (IAA), and abscisic acid (ABA) contents were measured in the leaves of the 45-day-old NT and *35S::AtCDF3* (L 2.3) plants grown under the control and salinity conditions (Figure 8; Figure S3). No differences were observed between the genotypes in the IP, IAA and ABA levels under the control conditions (Figure 8). Interestingly, active GA_1_ gibberellin content was higher (*P* < 0.05) in the transgenic plants. Higher active gibberellin levels have been related to growth promotion through increased source and sink activities (Iqbal et al., 2011). Remarkably, transcriptomic analyses of tomato *AtCDF3* overexpressing lines show an increased expression of the 3-hydroxy-3-methylglutaryl coenzyme A reductase (HMGR), catalyzing a rate-limiting step of isoprenoid synthesis through the mevalonate pathway (Pulido et al., 2012) and leading to the synthesis of gibberellins when the chloroplastic MEP pathway is limiting (Kasahara et al., 2002).

Under the salinity conditions, IAA and GA_1_ content lowered in both the NT and *35S::AtCDF3* plants. This decrease could be related to the reported drop in growth and production. ABA levels rose in both genotypes, but this increase was more prominent in the transgenic plants. Accordingly, ABA has been shown to alleviate salt stress in several species (Sah et al., 2016).

## Discussion

CDF transcription factors have been reported to participate in the regulation of processes related to plant growth and development, with special relevance on responses to abiotic stress conditions (Corrales et al., 2014, 2017; Fornara et al., 2015). In this work we have overexpressed Arabidopsis and tomato *CDF3* genes in tomato plants and assessed their precise impact on growth, yield and tomato fruit quality, under control and salt stress conditions. We reported that the plants overexpressing *CDF3* genes display increased growth and yield. Our results indicate that the higher biomass production is supported by higher photosynthetic rates and sucrose levels, but also by changes in carbon and nitrogen metabolism. Interestingly, despite photosynthesis, growth and yield were reduced by salinity; they were consistently higher in the transgenic plants than in the NT plants.

### Enhanced biomass production in plants overexpressing CDF3 transcription factors

In this work we have generated transgenic tomato plants overexpressing *CDF3* genes from tomato and Arabidopsis. Further, their phenotypic characterization shows that they present higher biomass production compared to the non-transformed plants (Figure 1). Since plant growth relies largely on photosynthesis and the production of carbohydrates and nitrogen compounds to provide energy and biomass, we performed combined gas exchange-chlorophyll fluorescence determinations and metabolomic analyses to gain insight into the physiological processes underlying the phenotypes observed.

Photosynthetic efficiency can be improved through the optimization of any of the limiting processes involved, including CO_2_ diffusion, light harvesting processes, CO_2_ fixation and assimilation, and carbon metabolism (Long et al., 2015). Several reports have indicated that stomatal conductance is critical for growth and yield, and greater conductance is related to higher fixation rate under non-limiting water conditions (Roche, 2015). Notably, *CDF3* overexpressing plants show higher stomatal conductance, thus improving CO_2_ diffusion to the mesophyll (Table 1). In addition, the transgenic plants display higher effective quantum yield of PSII (PhiPS2), related to the electron transport through PSII, and a higher maximum carboxylation rate of Rubisco. Taken together, these data indicate an improved photosynthetic capacity in the *CDF3* overexpressing plants.

Likewise, our results related to metabolite analyses in leaves show that the overexpression of the *CDF3* genes triggers manifold changes in carbon-skeleton production and nitrogen assimilation pathways (Figure 4). These plants contain higher sucrose levels, in agreement with the higher reported CO_2_ fixation rate. Starch levels remained almost unchanged, which suggests that C allocation favors the TCA anaplerotic flux in these plants. The marked increase in several amino acids, specially glutamine, glutamate, asparagine and GABA, indicates enhanced nitrogen assimilation. Both glutamine and glutamate are good indicators of nitrogen utilization (Stitt and Krapp, 1999; Foyer et al., 2006). The drop in citric acid content is in support of an increase of C skeletons, which diverge to amino acid synthesis through intermediate 2-oxoglutarate (2-OG), as expected from the increase in both the *GS2* and *AS* expression in transcriptomic data and the observed glutamate/glutamine and asparagine levels. As previously reported for the heterologous expression of *ZmDOF1* in Arabidopsis or rice, which modulates the expression of genes involved in C skeleton production for amino acid biosynthesis (Yanagisawa et al., 2004; Kurai et al., 2011), our findings also suggest the cooperative modification of carbon and nitrogen metabolism by the overexpression of CDF3 Dof-type TFs. In line with this, our study also revealed higher succinate and γ-amino butyric acid (GABA) content in the transgenic plants, and GABA is involved in nitrogen storage, among other roles (Shelp et al., 1999). The pathway that converts glutamate into succinate via GABA (GABA shunt) compensates the high flux rate from 2-oxoglutarate to amino acid metabolism (Araujo et al., 2012). Similarly, increased *PEPCK* expression is also observed, as previously associated with tissues in which the metabolism of nitrogenous compounds is enhanced (Chen et al., 2004).

The above-described metabolic changes have also been observed in Arabidopsis plants that overexpress *SlCDF3* (Corrales et al., 2014), which suggests that CDF3s may play a key conserved role in controlling the genes involved in carbon fixation and nitrogen assimilation.

### AtCDF3 regulates genes associated to plant growth, mostly involved in photosynthetic activity and C/N metabolism

Leaf transcriptomic analyses of *AtCDF3* overexpressing plants revealed increased expression levels of important target genes involved in photosynthesis and primary metabolism (Table 2; Tables S2–S5).

Noticeably, DnaJ chaperones DnaJ8, DnaJ11, and DnaJ20, and thioredoxins (CxxS1 and TRX3.1), are up-regulated in the plants that overexpress the *AtCDF3* gene. The higher *DnaJ* expression levels in the *35S::AtCDF3* tomato plants, which have also been reported in *35S::AtCDF3* Arabidopsis plants (Corrales et al., 2017) can be linked to the improved photosynthetic performance observed, since these proteins have been reported to be involved in the optimization of CO_2_ fixation, the stabilization of PSII complexes and the balancing of electron transfer reactions (Chen et al., 2010). Thioredoxins (TRXs) are members of sensing systems that monitor and maintain optimal redox conditions (König et al., 2012). Many essential plastid processes, such as carbon metabolism, nitrogen metabolism, lipid biosynthesis or protein folding, are regulated by TRXs (Serrato et al., 2013). Interestingly, a Dof TF (PsDOF7) has been reported to control TRX expression in response to sugars (Barajas-López et al., 2012), as observed in our work. Altogether, these data agree with the higher maximum carboxylation rate of Rubisco and the higher electron transport rate through the PSII observed under the control conditions in the plants that overexpress *CDF3*.

Key genes involved in C/N metabolism are also upregulated in the *CDF3* overexpressing plants. Among them, pyruvate kinase (*PK*) and PEP carboxykinase (*PEPCK*), participate in the glycolysis and gluconeogenesis pathways and probably confer greater metabolic flexibility to these plants. Interestingly, the *35S::AtCDF3* plants show higher expression of the glutamate synthase (*GS*) and glutamate decarboxylase (*GAD*) genes (Figure 3) in support of the altered metabolic profiles observed in these plants (Figure 4).

Additionally, other important genes related to plant growth are upregulated in the *CDF3* overexpressing plants (Table 2). Of special interest are different transcription factors, like PIFs, which are involved in growth promotion (Lucas and Prat, 2014) acting as central hubs of a regulatory programme that integrates internal (e.g., sucrose and gibberellins) and environmental signals (Leivar and Monte, 2014). It has to be noted that T6P and sucrose mediate the induction of PIFs (Liu et al., 2011) and both *T6P synthase* and *PIF1* genes are up-regulated in the *35S::CDF3* plants. Several PIF targets are implicated in cell elongation and the control of photosynthetic capacity (Lucas and Prat, 2014). Accordingly, Fornara et al. (2015) have related greater growth in Arabidopsis, mediated by PIF4, to the activity of CDFs.

Of the different hormones measured in the *CDF3* overexpressing plants we could detect higher levels of active GAs, which are known to induce the expression of cellulose synthase and expansin, involved in cell elongation and growth (Kordel and Kutschera, 1999; Lee and Kende, 2002). Interestingly, our transcriptomic analyses revealed higher expression levels of such genes: cellulose synthase, xyloglucan endo transglycosylase/hydrolase (*XTH*) and expansins (Table S2). Higher bioactive GA levels are reported to increase tomato fruit set and early fruit development through cell expansion (Mariotti et al., 2011; Ariizumi et al., 2013). Interestingly, an increased number of fruits with larger size were found in the plants that overexpress *CDF3* genes (Figure 5). Larger fruit size and sink demand have been linked to the activation of invertases induced by gibberellins (Roitsch and González, 2004; Albacete et al., 2014).

Altogether, our results highlight the potential role of *CDF3* in the regulation of the genes related to C/N metabolism, photosynthetic efficiency and growth.

### Overexpression of *SlCDF3* or *AtCDF3* genes results in changes in fruit composition associated with fruit quality improvement

The altered sugar and organic acid profiles in the *CDF3* overexpressing lines result in higher levels of sucrose equivalents, and in variables related to sweetness perception and overall acceptability of fruits (Baldwin et al., 1998; Bucheli et al., 1999). Increased GABA content was also observed in fruits, along with a considerable reduction in citrate content, which seems compatible with altered GABA shunt regulation, as pointed out in leaves. A similar profile has also been observed in GABA-rich tomato cultivars (Akihiro et al., 2008).

*CDF3* overexpressing lines show increased levels of malate, most likely due to the GABA shunt. Malate plays a key role in gluconeogenesis via PEPCK activity, which increases in the ripening stage of tomato fruits (Yin et al., 2010; Osorio et al., 2013) and would likely explain the mild rise in hexose content observed in some transgenic lines. An altered malate concentration has also been described to influence cellular redox status, and to result in altered carotenoid biosynthesis (Centeno et al., 2011), as observed in the fruits analyzed in the present study. Taken together, our results indicate that CDF3 overexpression induces changes in the composition of sugars and organic acids in fruits associated to the improvement in fruit quality, and highlights the interest of *CDF* genes in tomato breeding given their effects on fruit production and quality.

### Tomato plants overexpressing *CDF3* genes maintain increased growth and yield under salinity conditions

One of the major outputs of our work is the capacity of using CDF TFs in engineering plant metabolism for the improvement of tomato plants in their production and fruit quality under usual growing conditions. However, they are specially interesting when considering tomato growth and production under salinity stress. In Figure 5, although some reduction was observed compared to the control conditions, yield values under salinity (75 mM of NaCl) were higher in the transgenic plants than those of the NT plants. Previous work, based on the introgression of genes involved in the stress responses of tomato (Pandey et al., 2011), have shown their potential use to alleviate short-term applied drought and salinity stresses (usually measured as plant survival). Nevertheless, in very few cases an effect on crop yield stability has been proved (Albacete et al., 2014).

Increasing crop yield involves diverse physiological and biochemical processes, including photosynthesis, assimilate partitioning to reproductive structures and nitrogen uptake and metabolism (Sinclair et al., 2004). Under salinity, the improved responses of the *CDF3* overexpressing lines could be supported by the maintenance of greater carbon and nitrogen assimilation compared to NT plants. Moreover, these lines also display enhanced photosynthetic rates under this stress condition (Figure 1; Table 1). The minimal impact of salinity on the photosynthetic rate in the *35S::CDF3* plants could be explained by the maintained carboxylation rate of Rubisco and the electron transport rate of PSII (Table 1). In addition, the CO_2_ supply to the chloroplast in these plants appears ensured through high stomatal and mesophyll conductances. Furthermore, the increased photorespiration rate and the rise in the expression of genes related to protective activities and electron transport chain components, as observed in the transgenic plants, prevent oxidative damage under stress, reduce biochemical limitations and optimize photosynthesis (Voss et al., 2013).

The amount of sucrose, asparagine, phenylalanine and GABA also remained higher in the *CDF3* overexpressing plants (Figure 4). The change in the glutamine-glutamate/asparagine ratio was as expected (Lam et al., 1996) since energy-limited conditions favor nitrogen assimilation into asparagine. The increased amounts of the above-mentioned compounds have been related to the responses of plants to salt stress. Higher levels of asparagine and sucrose can improve osmotic adjustment under salinity. The importance of a functional GABA shunt for stress has also been observed in tomato under salinity (Zushi and Matsuzoe, 2006; Bao et al., 2015). A larger amount of phenylalanine can be related to phenolic compounds scavenge of free radicals and other oxidative species (Dicko et al., 2005).

Transcriptomic analyses of *CDF3* overexpressing plants grown under salinity, also showed an increased expression of certain TFs (Table 2), previously associated to abiotic stress tolerance (Agarwal et al., 2016; Hong et al., 2016). The fact that these TFs are also up-regulated in these plants under the control conditions, suggests that CDF3 is an upstream regulator of these genes, in accordance with similar results obtained in Arabidopsis plants that overexpress the *AtCDF3* gene (Corrales et al., 2017), and reinforces the role of the *CDF3* genes in the control of responses to stresses.

## Conclusion

The overexpression of *CDF3* genes from either Arabidopsis or tomato increases growth rate and, ultimately, the yield of tomato plants. The increased growth observed could be associated to higher photosynthetic rates, which lead to higher sucrose levels, and to changes in the C/N metabolism, including nitrogen assimilation. In addition, *CDF3* induced the up-regulation of genes involved in cell growth, photosynthesis efficiency, primary metabolism and stress responses. Our data revealed that CDFs are potential tools for improving tomato production under regular and salinity conditions.

## Author contributions

RM, JV, JM, and SN conceived and designed the experiments. BR, LC, JC, MS, JD, SP, AC, and SN performed the experiments. RM, JC, JF, JV, JM, and SN analyzed the data. All the authors contributed to the writing, discussion and approval of the final manuscript. BR, LC, and RM are to be considered equal joint first authors.

## Funding

This work has been supported by grants from the Instituto Nacional de Investigación y Tecnología Agraria y Alimentaria (Projects 2009-0004-C01 and 2012-0008-C01) and the Ministerio de Economía, Industria y Competitividad (Projects BFU2013-49665-EXP and BIO2014-53181-R). JD was supported by an INIA predoctoral fellowship.

### Conflict of interest statement

The authors declare that the research was conducted in the absence of any commercial or financial relationships that could be construed as a potential conflict of interest. The reviewer CJDO and handling Editor declared their shared affiliation, and the handling Editor states that the process nevertheless met the standards of a fair and objective review.
