# Supplementary material for: Ectopic Expression of CDF3 Genes in Tomato Enhances Biomass Production and Yield under Salinity Stress Conditions
Source: Front Plant Sci. 2017 May 3;8:660. doi: 10.3389/fpls.2017.00660 (PMC5414387; doi:10.3389/fpls.2017.00660)
Supplement: Supplementary file 1 [file Table1.DOCX]

| **Table S1. Primers used for Real-Time PCR analyses.** | | |
| --- | --- | --- |
| Gene | Primer sequence |  |
|  |  |  |
| *AtCDF3* | 5’-AGAAGGCCGGGTGCGTTCTG-3’ |  |
|  | 5’-ACCGGCTTTGCACATCGCCT-3’ |  |
| *SlCDF3* | 5’-AAAATCCTAAGACTCCATCAGAATCAG-3’ |  |
|  | 5’-GCTTGAGGGTCTTCCTCAGTTGA-3’ |  |
| *CXXS1* | 5’-GTTGAAGAACAGGGGACCAA-3’ |  |
|  | 5’-CATGAAAATCCCATGATTCCTT-3’ |  |
| *DNAJ8* | 5’-ACGGCTATGGGAATGATGAG-3’ |  |
|  | 5’-TCAAACGAATCCATGAAGCA-3’ |  |
| *GAD2* | 5’-CGTCGTTGTACCACCACTACGC-3’ |  |
|  | 5’-ACGCGAAAGTCGAGTGAACGG-3’ |  |
| *GI* | 5’-GCAACCATTGGAAAACAAAG-3’ |  |
|  | 5’-CAGACAGAAGCAAGGACATAAG-3’ |  |
| *GS2* | 5’-AGCTTCAGCCTCAAGGGTTGGC-3’ |  |
|  | 5’-CGCCCAGCTTCAAACATGGACC-3’ |  |
| *MYB44* | 5’-TCCGTACCCGTAACTTGTCC-3’ |  |
|  | 5’-CTGATTTGGGTGCAGCACTA-3’ |  |
| *PIF1* | 5’-GGCATTTTTCACGGTTGTCT-3’ |  |
|  | 5’-ACAATCGTCGATGCCCTAAC-3’ |  |
| *SOC1* | 5’-TTCTTGGGCTGTTCCCATTA-3’ |  |
|  | 5’-GATTGTTGTCTGTCCAAAAGATTC-3’ |  |
| *UBI3* | 5’-AAGCAATGGATGCTGAGGCT-3’ |  |
|  | 5’-GAAGGTGCCGTTGAATGACA-3’ |  |
