# Supplementary material for: Ectopic Expression of CDF3 Genes in Tomato Enhances Biomass Production and Yield under Salinity Stress Conditions
Source: Front Plant Sci. 2017 May 3;8:660. doi: 10.3389/fpls.2017.00660 (PMC5414387; doi:10.3389/fpls.2017.00660)
Supplement: Supplementary file 2 [file Table2.DOCX]

| **Table S2. Non-exhaustive list of up-regulated genes in the line 2.3 *35S::AtCDF3* plants in control conditions.** Leaf transcriptomic analysis performed in 45-day-old plants grown in hydroponic culture. | | |
| --- | --- | --- |
| GeneID | P-value | Gene name |
| Solyc03g121540.2.1 | 0 | beta-galactosidase precursor |
| Solyc03g117590.2.1 | 0 | heat shock protein binding protein |
| Solyc09g092110.2.1 | 0 | light-regulated protein-like |
| Solyc09g092430.2.1 | 0 | selenium-binding protein 2-like |
| Solyc03g006490.2.1 | 0 | expressed predominantly in leaves |
| Solyc01g087800.2.1 | 0 | subtilisin-like protease-like |
| Solyc11g044910.1.1 | 0 | Xyl1 protein precursor |
| Solyc06g073090.2.1 | 0 | chloroplast-specific ribosomal protein |
| Solyc08g078870.1.1 | 0 | 14 kDa proline-rich protein DC2.15-like |
| Solyc02g077880.2.1 | 0 | auxin repressed/dormancy associated protein |
| Solyc04g071140.2.1 | 0 | histidine decarboxylase-like isoform 1 |
| Solyc12g094620.1.1 | 0 | catalase isozyme 1 |
| Solyc09g097770.2.1 | 0 | cell wall protein precursor |
| Solyc03g114150.2.1 | 8,49E-281 | aldehyde dehydrogenase family 2 member B4, mitochondrial-like |
| Solyc10g005100.2.1 | 1,24E-279 | plasma membrane cation-binding protein 1-like isoform 1 |
| Solyc12g100060.1.1 | 7,14E-223 | zinc finger A20 stress-associated protein 5-like |
| Solyc01g080020.2.1 | 8,45E-212 | basic 7S globulin-like |
| Solyc12g044330.1.1 | 1,66E-202 | aquaporin TIP2-1-like |
| Solyc01g111880.2.1 | 1,65E-183 | tyrosine-protein kinase transforming protein Fes-like isoform 2 |
| Solyc03g006030.2.1 | 7,43E-177 | LRR receptor-like serine/threonine-protein kinase GSO1-like |
| Solyc04g076880.2.1 | 7,24E-173 | phosphoenolpyruvate carboxykinase |
| Solyc06g068500.2.1 | 8,18E-171 | chaperone protein dnaJ 8, chloroplastic-like |
| Solyc10g083880.1.1 | 7,77E-151 | aquaporin TIP1-1-like |
| Solyc12g008840.1.1 | 1,32E-149 | beta-galactosidase |
| Solyc12g005860.1.1 | 1,08E-142 | aconitate hydratase, cytoplasmic-like isoform 2 |
| Solyc08g065610.2.1 | 4,80E-139 | vacuolar-processing enzyme-like |
| Solyc03g007810.2.1 | 5,24E-122 | plastidial pyruvate kinase 2-like |
| Solyc07g007930.2.1 | 8,58E-122 | galactinol-sucrose galactosyltransferase 6-like |
| Solyc05g009790.1.1 | 8,71E-122 | AP2/ERF domain-containing transcription repressor TEM1-like |
| Solyc03g115650.2.1 | 4,89E-120 | eukaryotic translation initiation factor 5A-1 |
| Solyc08g077900.2.1 | 5,27E-114 | expansin-like B1-like |
| Solyc04g007000.1.1 | 5,48E-107 | RAV1 |
| Solyc08g077330.2.1 | 1,18E-102 | expansin-like B1-like |
| Solyc12g008650.1.1 | 3,67E-101 | myo-inositol oxygenase |
| Solyc01g106620.2.1 | 9,26E-94 | PR1 protein precursor |
| Solyc12g099970.1.1 | 1,13E-91 | SNF1 kinase complex anchoring protein |
| Solyc06g008030.2.1 | 1,06E-86 | transcription factor PIF1-like isoform 1 |
| Solyc09g092520.2.1 | 3,39E-83 | brassinosteroid-regulated protein BRU1 |
| Solyc04g017690.2.1 | 2,23E-80 | protein EARLY RESPONSIVE TO DEHYDRATION 15-like |
| Solyc01g111980.2.1 | 4,22E-76 | lysine histidine transporter-like 8-like |
| Solyc01g010050.2.1 | 1,86E-74 | S-adenosylmethionine decarboxylase 3 |
| Solyc08g080670.1.1 | 2,70E-74 | PR5-like protein precursor |
| Solyc03g111310.2.1 | 1,77E-73 | SNF1-related protein kinase regulatory subunit gamma-1-like |
| Solyc02g070280.2.1 | 3,91E-71 | cationic amino acid transporter 1-like |
| Solyc03g120980.2.1 | 1,40E-70 | ABC transporter G family member 36-like |
| Solyc03g005450.2.1 | 3,59E-69 | cellulose synthase-like protein G2-like |
| Solyc03g115700.2.1 | 3,93E-69 | SNF1-related protein kinase |
| Solyc06g007180.2.1 | 4,82E-66 | asparagine synthetase |
| Solyc03g006360.2.1 | 2,05E-61 | auxin-repressed 12.5 kDa protein-like |
| Solyc11g020670.1.1 | 5,93E-60 | TCP transcription factor 12 |
| Solyc04g077860.2.1 | 1,45E-58 | sigma factor SigB regulation protein RsbQ-like |
| Solyc06g066370.2.1 | 5,60E-58 | probable WRKY transcription factor 33-like |
| Solyc07g066330.2.1 | 1,10E-57 | NAC domain-containing protein 21/22-like |
| Solyc06g054540.2.1 | 2,91E-57 | RING finger and CHY zinc finger domain-containing protein 1-like |
| Solyc03g019820.2.1 | 6,05E-55 | tonoplast intrinsic protein 3;2 |
| Solyc12g056650.1.1 | 9,22E-55 | protein GIGANTEA-like |
| Solyc02g072150.2.1 | 3,23E-52 | alpha,alpha-trehalose-phosphate synthase |
| Solyc11g008440.1.1 | 4,84E-52 | vacuolar amino acid transporter 1-like |
| Solyc07g006500.2.1 | 1,79E-51 | trehalose-6-phosphate synthase |
| Solyc01g080870.2.1 | 1,96E-49 | nitrate transporter 1.5-like |
| Solyc04g081530.1.1 | 4,52E-49 | DNAJ-like protein |
| Solyc10g017960.1.1 | 1,21E-46 | F-box protein PP2-A13-like |
| Solyc04g076990.2.1 | 6,90E-46 | receptor-like protein kinase HAIKU2-like |
| Solyc05g046290.2.1 | 1,23E-44 | xyloglucan endotransglucosylase/hydrolase protein 23-like |
| Solyc04g071990.2.1 | 6,23E-44 | protein GIGANTEA-like |
| Solyc09g010400.2.1 | 7,12E-44 | probable histone H2A.1-like [Solanum lycopersicum] |
| Solyc08g080660.1.1 | 2,59E-43 | osmotin-like protein OSML15-like |
| Solyc01g005210.2.1 | 2,69E-43 | alpha,alpha-trehalose-phosphate synthase [UDP-forming] 6-like |
| Solyc05g007070.2.1 | 5,83E-41 | alpha-amylase 3, chloroplastic-like |
| Solyc01g097270.2.1 | 3,70E-38 | wound-induced protein WIN2 |
| Solyc02g092580.2.1 | 2,79E-35 | peroxidase 73-like |
| Solyc01g109170.2.1 | 2,81E-35 | cold-regulated 413 plasma membrane protein 2-like isoform 1 |
| Solyc02g084570.2.1 | 5,63E-35 | cytochrome P450 84A1-like |
| Solyc05g007770.2.1 | 7,94E-34 | NAC transcription factor 29-like |
| Solyc01g112000.2.1 | 1,14E-33 | expansin-like protein precursor |
| Solyc04g071120.2.1 | 1,71E-32 | RAF proto-oncogene serine/threonine-protein kinase-like |
| Solyc12g010900.1.1 | 4,71E-32 | F-box protein ORE9-like |
| Solyc08g075420.2.1 | 6,71E-32 | zinc finger CCHC domain-containing protein At4g19190-like |
| Solyc12g042950.1.1 | 3,79E-31 | plastidic ATP/ADP-transporter-like |
| Solyc00g009110.2.1 | 4,93E-31 | inositol-1,4,5-triphosphate-5-phosphatase |
| Solyc06g073190.2.1 | 4,97E-31 | fructokinase-2 |
| Solyc06g007430.1.1 | 2,14E-30 | CBL-interacting protein kinase 2-like |
| Solyc06g082010.2.1 | 6,54E-30 | zinc finger CCCH domain-containing protein 66-like isoform 1 |
| Solyc04g078420.1.1 | 1,88E-29 | transcription factor MYB44-like |
| Solyc10g078920.1.1 | 3,54E-29 | thioredoxin-like 3-1, chloroplastic-like |
| Solyc08g021820.2.1 | 5,18E-29 | IAA29 |
| Solyc10g055810.1.1 | 3,54E-27 | basic 30 kDa endochitinase precursor |
| Solyc08g079420.2.1 | 9,02E-27 | cytochrome P450 93A1-like |
| Solyc01g092950.2.1 | 1,07E-26 | MADS-box protein SOC1-like |
| Solyc04g007070.2.1 | 1,74E-26 | putative late blight resistance protein homolog R1B-23-like |
| Solyc09g008430.2.1 | 4,89E-25 | RING finger and CHY zinc finger domain-containing protein 1-like |
| Solyc03g032040.2.1 | 5,00E-25 | monosaccharide-sensing protein 2-like |
| Solyc02g068300.2.1 | 6,10E-25 | L-type lectin-domain containing receptor kinase S.5-like |
| Solyc02g080070.2.1 | 8,10E-25 | cysteine-rich receptor-like protein kinase 10-like |
| Solyc07g040960.1.1 | 1,03E-23 | salt responsive protein 2 |
| Solyc09g092260.2.1 | 2,32E-23 | chaperone protein dnaJ 20, chloroplastic-like isoform 2 |
| Solyc03g114950.2.1 | 4,01E-23 | ABC transporter B family member 25-like |
| Solyc06g071050.2.1 | 7,69E-23 | hypersensitive-induced response protein 1-like |
| Solyc08g080650.1.1 | 2,53E-22 | pathogenesis-related protein PR P23 |
| Solyc05g005750.2.1 | 1,31E-20 | alpha,alpha-trehalose-phosphate synthase 9-like |
| Solyc02g070910.1.1 | 4,37E-20 | LRR receptor-like serine/threonine-protein kinase FLS2-like |
| Solyc04g005610.2.1 | 7,51E-20 | NAC transcription factor 29-like |
| Solyc04g050620.2.1 | 1,98E-18 | cytochrome P450 71A1-like |
| Solyc09g009590.1.1 | 7,37E-18 | stress-associated protein 8 |
| Solyc07g053740.1.1 | 1,07E-17 | ethylene-responsive transcription factor 4-like |
| Solyc06g050500.2.1 | 3,18E-17 | abscisic acid receptor PYL6-like |
| Solyc04g071890.2.1 | 3,27E-17 | peroxidase 12-like |
| Solyc06g071810.1.1 | 6,43E-17 | leu-rich repeat receptor-like ser/thre/tyr-protein kinase SOBIR1-like |
| Solyc09g009100.2.1 | 6,45E-17 | heat stress transcription factor A3 |
| Solyc12g087830.1.1 | 1,72E-16 | agamous-like MADS-box protein AGL31-like |
| Solyc01g010770.2.1 | 3,18E-16 | hypersensitive-induced response protein 1-like |
| Solyc09g011560.2.1 | 3,43E-16 | probable glutathione S-transferase-like |
| Solyc12g098520.1.1 | 3,74E-16 | heat stress transcription factor A-5-like |
| Solyc02g071740.2.1 | 3,80E-16 | probable serine/threonine-protein kinase drkD-like |
| Solyc01g056850.2.1 | 6,73E-16 | CTP synthase-like |
| Solyc08g080630.2.1 | 1,36E-15 | ethylene-responsive proteinase inhibitor 1 precursor |
| Solyc07g065970.1.1 | 1,52E-15 | chaperone protein dnaJ 11, chloroplastic-like |
| Solyc02g062890.1.1 | 1,76E-15 | probable polyol transporter 6-like |
| Solyc09g082830.2.1 | 3,97E-15 | protein argonaute 10-like |
| Solyc12g096830.1.1 | 7,00E-15 | UDP-glycosyltransferase 74E2-like |
| Solyc12g017630.1.1 | 1,05E-14 | ribosomal RNA small subunit methyltransferase NEP1-like |
| Solyc07g064620.1.1 | 1,08E-14 | protein translation factor SUI1 homolog isoform 1 |
| Solyc09g059030.2.1 | 1,10E-14 | quinone-oxidoreductase homolog, chloroplastic-like isoform 1 |
| Solyc09g090980.2.1 | 1,43E-14 | pathogenesis-related protein STH-2-like |
| Solyc07g052790.1.1 | 2,42E-14 | protein SUPPRESSOR OF npr1-1, CONSTITUTIVE 1-like |
| Solyc09g005480.2.1 | 2,65E-14 | F-box protein At2g40925-like |
| Solyc06g068570.2.1 | 3,35E-14 | AP2-like ethylene-responsive transcription factor At1g16060-like |
| Solyc03g113910.2.1 | 3,46E-14 | gibberellin-regulated protein 10-like |
| Solyc02g079960.2.1 | 4,82E-14 | thioredoxin-like protein CXXS1-like |
