# Supplementary material for: Ectopic Expression of CDF3 Genes in Tomato Enhances Biomass Production and Yield under Salinity Stress Conditions
Source: Front Plant Sci. 2017 May 3;8:660. doi: 10.3389/fpls.2017.00660 (PMC5414387; doi:10.3389/fpls.2017.00660)
Supplement: Supplementary file 3 [file Table3.DOCX]

| **Table S3. Non-exhaustive list of down-regulated genes in the line 2.3 *35S::AtCDF3* plants in control conditions.** Leaf transcriptomic analysis performed in 45-day-old plants grown in hydroponic culture | | |
| --- | --- | --- |
| GeneID | P-value | Gene name |
| Solyc11g012700.1.1 | 0 | oligopeptide transporter 3-like |
| Solyc07g043390.2.1 | 0 | cellulose synthase-like protein G1-like |
| Solyc04g054740.2.1 | 0 | inositol-3-phosphate synthase |
| Solyc08g074620.1.1 | 0 | polyphenol oxidase E, chloroplastic-like isoform 1 |
| Solyc02g086820.2.1 | 0 | carbonic anhydrase |
| Solyc03g098780.1.1 | 4,99E-202 | serine protease inhibitor 1-like isoform 1 |
| Solyc11g021060.1.1 | 9,98E-175 | proteinase inhibitor type-2 TR8 precursor |
| Solyc10g005400.2.1 | 4,25E-174 | inositol oxygenase 1-like |
| Solyc11g066670.1.1 | 6,23E-160 | zeatin O-glucosyltransferase-like |
| Solyc11g028100.1.1 | 1,36E-145 | elongation factor 1-gamma 2-like isoform 3 |
| Solyc02g086650.2.1 | 4,07E-121 | phosphoenolpyruvate/phosphate translocator 2 |
| Solyc02g092800.2.1 | 1,34E-110 | metal transporter |
| Solyc05g014380.2.1 | 1,43E-88 | ABC transporter C family member 3-like |
| Solyc02g080220.2.1 | 2,31E-75 | pectinesterase/pectinesterase inhibitor 18-like isoform 1 |
| Solyc10g008410.1.1 | 6,85E-64 | E3 ubiquitin-protein ligase RMA1H1-like isoform 1 |
| Solyc03g111690.2.1 | 7,31E-58 | probable pectate lyase 18-like |
| Solyc02g077420.2.1 | 1,66E-57 | phospholipase A1-II 1-like isoform 1 |
| Solyc05g007830.2.1 | 1,40E-46 | expansin-A1 |
| Solyc09g010530.2.1 | 3,95E-46 | cation/H(+) antiporter 20-like |
| Solyc05g009500.2.1 | 1,83E-40 | probable nitrite transporter At1g68570-like |
| Solyc02g082260.2.1 | 2,93E-40 | 3-hydroxy-3-methylglutaryl-coenzyme A reductase 1 |
| Solyc08g080170.2.1 | 5,18E-40 | hydroxymethylglutaryl-CoA synthase-like |
| Solyc10g085870.1.1 | 1,05E-39 | UDP-glycosyltransferase 73C3-like |
| Solyc06g019170.2.1 | 9,57E-36 | delta-1-pyrroline-5-carboxylate synthase-like |
| Solyc11g010850.1.1 | 1,01E-34 | 1-deoxy-D-xylulose-5-phosphate synthase 2 |
| Solyc08g082980.2.1 | 1,91E-32 | serine/threonine-protein kinase WNK4-like isoform 1 |
| Solyc04g064770.1.1 | 1,19E-31 | zinc finger CCCH domain-containing protein 55-like |
| Solyc10g086180.1.1 | 7,85E-31 | phenylalanine ammonia-lyase-like |
| Solyc04g054990.2.1 | 1,31E-28 | lipoxygenase homology domain-containing protein 1-like |
| Solyc01g105450.2.1 | 2,22E-27 | ABC transporter G family member 11-like |
| Solyc09g083440.2.1 | 4,06E-27 | wound-induced proteinase inhibitor 1-like |
| Solyc05g053550.2.1 | 2,49E-22 | chalcone synthase 2 |
| Solyc10g079640.1.1 | 3,74E-22 | IAA-amino acid hydrolase ILR1-like 6-like isoform 1 |
| Solyc05g017760.2.1 | 1,25E-19 | acetyl-CoA acetyltransferase, cytosolic 1-like |
| Solyc01g111570.2.1 | 8,22E-19 | receptor-like ser/thre-protein kinase At5g57670-like |
| Solyc09g084490.2.1 | 4,11E-18 | wound-induced proteinase inhibitor 1-like |
| Solyc06g050900.2.1 | 3,16E-17 | probable peptide/nitrate transporter At2g40460-like |
| Solyc07g006570.2.1 | 4,80E-15 | ribonuclease 3-like |
