# Supplementary material for: Ectopic Expression of CDF3 Genes in Tomato Enhances Biomass Production and Yield under Salinity Stress Conditions
Source: Front Plant Sci. 2017 May 3;8:660. doi: 10.3389/fpls.2017.00660 (PMC5414387; doi:10.3389/fpls.2017.00660)
Supplement: Supplementary file 4 [file Table4.DOCX]

| **Table S4.** **Non-exhaustive list of up-regulated genes in the line 2.3 *35S::AtCDF3* plants under salinity conditions.** Thirty-day-old plants grown in hydroponic culture were subjected to moderate salinity (75 mM NaCl). Leaf transcriptomic analysis was performed after 15 days. | | | |
| --- | --- | --- | --- |
| GeneID | P-value | Gene name | |
| Solyc08g065610.2.1 | 0 | | vacuolar-processing enzyme-like |
| Solyc00g174340.1.1 | 0 | | pathogenesis-related leaf protein 6 precursor |
| Solyc05g050120.2.1 | 0 | | cytosolic NADP-malic enzyme |
| Solyc12g094620.1.1 | 0 | | catalase isozyme 1 |
| Solyc09g097770.2.1 | 0 | | cell wall protein precursor |
| Solyc04g080960.2.1 | 0 | | cysteine proteinase 15A |
| Solyc12g088670.1.1 | 0 | | low-temperature-induced cysteine proteinase precursor |
| Solyc10g055810.1.1 | 3,17E-293 | | basic 30 kDa endochitinase precursor |
| Solyc10g009410.1.1 | 7,14E-258 | | protein ASPARTIC PROTEASE IN GUARD CELL 1-like |
| Solyc09g091660.2.1 | 8,09E-204 | | pleiotropic drug resistance protein 1-like |
| Solyc07g049530.2.1 | 3,76E-193 | | 1-aminocyclopropane-1-carboxylate oxidase 1 |
| Solyc08g080650.1.1 | 6,22E-173 | | pathogenesis-related protein PR P23 |
| Solyc03g122350.2.1 | 3,05E-147 | | flavonoid 3'-monooxygenase-like |
| Solyc08g016270.1.1 | 6,55E-145 | | LRR receptor-like serine/threonine-protein kinase |
| Solyc01g006300.2.1 | 8,27E-142 | | peroxidase precursor [Solanum lycopersicum] |
| Solyc10g055800.1.1 | 5,14E-141 | | endochitinase 3-like |
| Solyc04g074000.2.1 | 5,36E-133 | | LRR receptor-like serine/threonine-protein kinase |
| Solyc08g079430.2.1 | 1,26E-132 | | primary amine oxidase |
| Solyc01g107820.2.1 | 1,16E-127 | | anthocyanin 3'-O-beta-glucosyltransferase |
| Solyc09g090980.2.1 | 1,54E-122 | | pathogenesis-related protein STH-2-like |
| Solyc09g090730.1.1 | 2,01E-122 | | ammonium transporter 1 member 1-like |
| Solyc01g097270.2.1 | 1,29E-114 | | wound-induced protein WIN2 |
| Solyc03g006030.2.1 | 3,58E-102 | | LRR receptor-like serine/threonine-protein kinase |
| Solyc02g092670.1.1 | 5,93E-96 | | subtilisin-like protease-like |
| Solyc04g071800.2.1 | 2,15E-94 | | cytochrome P450 71A1-like |
| Solyc02g082920.2.1 | 7,78E-94 | | acidic 26 kDa endochitinase precursor |
| Solyc03g111800.2.1 | 5,54E-89 | | leucine-rich repeat receptor-like serine/threonine/tyrosine-protein kinase SOBIR1-like |
| Solyc11g020280.1.1 | 1,47E-83 | | receptor-like protein kinase HAIKU2-like |
| Solyc12g006460.1.1 | 6,29E-75 | | ent-kaurenoic acid oxidase 2-like isoform 1 |
| Solyc02g080070.2.1 | 1,92E-71 | | cysteine-rich receptor-like protein kinase 10-like |
| Solyc04g071890.2.1 | 3,81E-71 | | peroxidase 12-like |
| Solyc12g008900.1.1 | 8,07E-69 | | cytokinin dehydrogenase 3 |
| Solyc09g082810.2.1 | 4,08E-68 | | glycine-rich protein |
| Solyc07g041920.2.1 | 4,23E-68 | | cysteine proteinase 3-like |
| Solyc03g113220.2.1 | 7,83E-65 | | hypersensitive-induced response protein 1 isoform 2 |
| Solyc03g007230.2.1 | 2,03E-63 | | protein phosphatase 2C 8-like |
| Solyc00g009020.2.1 | 5,12E-63 | | ATP synthase 24 kDa subunit, mitochondrial-like |
| Solyc03g119080.2.1 | 1,10E-61 | | beta-mannosidase enzyme precursor |
| Solyc09g007010.1.1 | 9,53E-60 | | pathogenesis-related leaf protein 4 precursor |
| Solyc06g071810.1.1 | 2,93E-59 | | leucine-rich repeat receptor-like serine/threonine/tyrosine-protein kinase SOBIR1-like |
| Solyc08g079420.2.1 | 9,39E-59 | | cytochrome P450 93A1-like |
| Solyc08g006330.2.1 | 4,57E-57 | | UDP-glycosyltransferase 74E1-like |
| Solyc07g065380.2.1 | 6,59E-57 | | zinc transporter-like precursor |
| Solyc07g053830.2.1 | 1,19E-56 | | ADP,ATP carrier protein 3, mitochondrial-like |
| Solyc09g090070.1.1 | 2,79E-55 | | inorganic phosphate transporter |
| Solyc12g100030.1.1 | 3,61E-55 | | receptor-like protein 12-like |
| Solyc02g065190.2.1 | 5,92E-55 | | geraniol 8-hydroxylase-like |
| Solyc04g007980.2.1 | 3,12E-53 | | 1-aminocyclopropane-1-carboxylate oxidase |
| Solyc08g082640.2.1 | 7,67E-53 | | cellulose synthase-like protein G3-like |
| Solyc01g094910.2.1 | 1,64E-51 | | ferric-chelate reductase |
| Solyc03g095650.2.1 | 2,67E-51 | | MLO-like protein 2-like |
| Solyc12g042480.1.1 | 1,43E-50 | | flavonoid 3',5'-hydroxylase 2-like |
| Solyc02g077040.2.1 | 8,39E-49 | | phytophthora-inhibited protease 1 |
| Solyc05g005570.2.1 | 1,32E-47 | | polygalacturonase-1 non-catalytic subunit beta-like |
| Solyc07g043230.2.1 | 6,94E-47 | | zinc transporter 5-like |
| Solyc04g071780.2.1 | 1,65E-46 | | flavonoid 3'-monooxygenase-like |
| Solyc01g105660.2.1 | 5,44E-46 | | probable 2-oxoglutarate/Fe(II)-dependent dioxygenase |
| Solyc10g084960.1.1 | 9,80E-45 | | glutathione S-transferase-like |
| Solyc12g089220.1.1 | 3,33E-44 | | bifunctional nuclease 1-like |
| Solyc05g007510.2.1 | 1,57E-43 | | RNA-directed RNA polymerase |
| Solyc07g006370.1.1 | 2,64E-42 | | cation/calcium exchanger 1-like |
| Solyc05g055310.2.1 | 3,86E-42 | | copper chaperone |
| Solyc11g017270.1.1 | 3,20E-41 | | leucine-rich repeat receptor protein kinase EXS-like |
| Solyc02g070280.2.1 | 6,89E-41 | | cationic amino acid transporter 1-like |
| Solyc01g090340.2.1 | 1,55E-40 | | ethylene-responsive transcription factor 1-like |
| Solyc03g111310.2.1 | 2,36E-39 | | SNF1-related protein kinase regulatory subunit gamma |
| Solyc04g079640.2.1 | 7,63E-38 | | cytochrome P450 82A4-like |
| Solyc12g008500.1.1 | 4,36E-37 | | leucine-rich repeat receptor protein kinase EXS-like |
| Solyc11g020670.1.1 | 5,29E-37 | | TCP transcription factor 12 |
| Solyc01g087810.2.1 | 2,67E-36 | | subtilisin-like protease-like |
| Solyc09g090080.1.1 | 1,70E-35 | | inorganic phosphate transporter 1-7-like |
| Solyc06g076750.2.1 | 4,11E-35 | | peptide transporter PTR1-like |
| Solyc06g008030.2.1 | 1,00E-34 | | transcription factor PIF1-like isoform 1 |
| Solyc10g054900.1.1 | 4,09E-32 | | proline-rich protein 4-like |
| Solyc02g082080.1.1 | 4,35E-32 | | copper transporter 5-like |
| Solyc12g096190.1.1 | 5,00E-32 | | tryptophan synthase beta chain 2-like |
| Solyc08g067340.2.1 | 6,17E-32 | | WRKY transcription factor 40-like |
| Solyc02g071210.2.1 | 1,52E-31 | | cold-regulated inner chloroplast membrane protein 2 |
| Solyc05g050380.2.1 | 3,92E-31 | | cyclic nucleotide-gated ion channel 1-like |
| Solyc07g066010.2.1 | 2,38E-30 | | amino acid permease 7-like |
| Solyc00g009110.2.1 | 6,39E-30 | | inositol-1,4,5-triphosphate-5-phosphatase |
| Solyc09g089930.1.1 | 1,21E-29 | | transcription factor TSRF1 |
| Solyc11g011240.1.1 | 8,93E-28 | | geranylgeranyl pyrophosphate synthase 1 |
| Solyc08g079900.1.1 | 1,42E-27 | | pathogenesis related protein P69G |
| Solyc07g052790.1.1 | 1,53E-27 | | protein SUPPRESSOR OF npr1-1, CONSTITUTIVE 1-like |
| Solyc03g113420.2.1 | 1,59E-27 | | peptide/nitrate transporter At5g62680-like |
| Solyc07g041910.2.1 | 3,71E-26 | | cysteine proteinase 3-like |
| Solyc06g074090.2.1 | 3,75E-26 | | 7-dehydrocholesterol reductase-like |
| Solyc00g050130.1.1 | 3,80E-26 | | zeatin O-glucosyltransferase-like |
| Solyc08g077330.2.1 | 8,67E-26 | | expansin-like B1-like |
| Solyc04g082030.1.1 | 1,77E-24 | | ornithine decarboxylase |
| Solyc03g117810.2.1 | 3,42E-24 | | ABC transporter I family member 17-like |
| Solyc01g006550.2.1 | 1,34E-23 | | receptor-like protein 12-like |
| Solyc09g007940.2.1 | 3,25E-23 | | adenosine kinase 2-like |
| Solyc01g098590.2.1 | 5,85E-23 | | broad-range acid phosphatase DET1-like |
| Solyc02g071470.2.1 | 2,48E-22 | | protein SRG1-like |
| Solyc10g081310.1.1 | 7,45E-22 | | 14 kDa zinc-binding protein-like |
| Solyc02g069800.1.1 | 1,18E-21 | | carboxylesterase 17-like isoform 1 |
| Solyc03g082690.2.1 | 1,46E-21 | | U-box domain-containing protein 43 |
| Solyc03g006360.2.1 | 4,77E-21 | | auxin-repressed 12.5 kDa protein-like |
| Solyc11g011180.1.1 | 6,88E-21 | | LRR receptor-like serine/threonine-protein kinase |
| Solyc01g106620.2.1 | 1,33E-20 | | PR1 protein precursor |
| Solyc02g032660.2.1 | 1,50E-20 | | protein TRANSPARENT TESTA 12-like |
| Solyc02g072470.2.1 | 1,92E-20 | | LRR receptor-like serine/threonine-protein kinase |
| Solyc01g008510.2.1 | 2,30E-20 | | photosystem II 5 kDa protein, chloroplastic-like |
| Solyc07g066330.2.1 | 3,44E-20 | | NAC domain-containing protein 21/22-like |
| Solyc12g045020.1.1 | 4,47E-20 | | cytochrome P450 84A1-like isoform 2 |
| Solyc01g009810.2.1 | 7,31E-20 | | somatic embryogenesis receptor kinase 2-like |
| Solyc02g086850.1.1 | 7,31E-20 | | RNA exonuclease 4-like |
| Solyc01g067020.2.1 | 9,56E-20 | | atypical receptor-like kinase 1 precursor |
| Solyc09g059040.2.1 | 3,67E-19 | | quinone-oxidoreductase homolog, chloroplastic-like |
| Solyc04g040130.1.1 | 5,94E-19 | | omega-6 fatty acid desaturase, endoplasmic reticulum |
| Solyc09g091550.2.1 | 5,97E-19 | | S-adenosyl-L-methionine: salicylic acid carboxyl methyltransferase |
| Solyc02g092750.2.1 | 1,75E-18 | | protein phosphatase 2C 78-like |
| Solyc04g074030.2.1 | 3,64E-18 | | LRR receptor-like serine/threonine-protein kinase |
| Solyc02g087110.2.1 | 1,18E-17 | | alpha-dioxygenase 1-like |
| Solyc03g113430.2.1 | 1,78E-17 | | peptide/nitrate transporter At5g62680-like |
| Solyc07g005440.1.1 | 1,98E-17 | | CBL-interacting serine/threonine-protein kinase 6-like |
| Solyc11g017280.1.1 | 4,62E-17 | | LRR receptor-like serine/threonine-protein kinase |
| Solyc09g011560.2.1 | 5,47E-17 | | glutathione S-transferase-like |
| Solyc05g005920.2.1 | 6,04E-17 | | peptide transporter At1g52190-like |
| Solyc12g087830.1.1 | 6,29E-17 | | agamous-like MADS-box protein AGL31-like |
| Solyc04g071990.2.1 | 7,89E-17 | | protein GIGANTEA-like |
| Solyc05g010340.2.1 | 1,10E-16 | | uridine-cytidine kinase C-like |
| Solyc05g018230.2.1 | 1,83E-16 | | sugar transporter, putative |
| Solyc02g071090.2.1 | 5,18E-16 | | purine permease 9-like |
| Solyc07g055710.2.1 | 6,64E-16 | | heat stress transcription factor A-4b-like |
| Solyc05g050360.2.1 | 1,52E-15 | | cyclic nucleotide-gated ion channel 1-like |
| Solyc03g120110.2.1 | 1,77E-15 | | G-type lectin S-receptor-like ser/thr-protein kinase |
| Solyc05g009610.1.1 | 2,17E-15 | | carboxylesterase 6-like |
| Solyc03g114960.2.1 | 4,42E-15 | | RING finger protein B-like |
| Solyc08g007790.2.1 | 5,91E-15 | | 3-hydroxy-3-methylglutaryl coenzyme A synthase |
| Solyc09g083200.2.1 | 7,31E-15 | | lysM domain receptor-like kinase 4-like |
| Solyc06g063210.2.1 | 1,97E-14 | | glutamate receptor 2.5 |
| Solyc09g072700.2.1 | 1,60E-13 | | peroxidase 44-like isoform 1 |
| Solyc03g114940.2.1 | 3,53E-12 | | cytochrome P450 78A4-like |
