# Supplementary material for: Ectopic Expression of CDF3 Genes in Tomato Enhances Biomass Production and Yield under Salinity Stress Conditions
Source: Front Plant Sci. 2017 May 3;8:660. doi: 10.3389/fpls.2017.00660 (PMC5414387; doi:10.3389/fpls.2017.00660)
Supplement: Supplementary file 5 [file Table5.DOCX]

| **Table S5.** **Non-exhaustive list of down-regulated genes in the line 2.3 *35S::AtCDF3* plants under salinity conditions.** Thirty-day-old plants grown in hydroponic culture were subjected to moderate salinity (75 mM NaCl). Leaf transcriptomic analysis was performed after 15 days. | | |
| --- | --- | --- |
| GeneID | P-value | Gene name |
| Solyc03g098790.1.1 | 0 | Cathepsin D Inhibitor |
| Solyc03g121540.2.1 | 0 | beta-galactosidase precursor |
| Solyc10g007600.2.1 | 0 | peroxisomal (S)-2-hydroxy-acid oxidase GLO5 isoform 1 |
| Solyc02g070980.1.1 | 0 | Chlorophyll a-b binding protein 1B, chloroplastic |
| Solyc04g054740.2.1 | 0 | inositol-3-phosphate synthase |
| Solyc08g074630.1.1 | 1,56E-268 | polyphenol oxidase F, chloroplastic-like |
| Solyc05g007830.2.1 | 4,40E-193 | expansin-A1 |
| Solyc01g111660.2.1 | 1,17E-186 | plasmamembrane intrinsic protein 2;8 |
| Solyc06g049050.2.1 | 3,36E-178 | expansin precursor |
| Solyc03g006490.2.1 | 4,30E-170 | expressed predominantly in leaves |
| Solyc07g052480.2.1 | 2,60E-159 | isocitrate lyase |
| Solyc11g066390.1.1 | 2,89E-152 | superoxidase dismutase |
| Solyc07g055060.2.1 | 1,86E-144 | PEPC, housekeeping isozyme-like |
| Solyc03g111120.2.1 | 2,37E-132 | malate synthase, glyoxysomal-like |
| Solyc03g111690.2.1 | 4,49E-117 | probable pectate lyase 18-like |
| Solyc09g082760.2.1 | 6,79E-111 | aspartic proteinase oryzasin-1-like |
| Solyc03g006100.2.1 | 3,89E-100 | LRR receptor-like ser/thr-protein kinase GSO1-like |
| Solyc12g044330.1.1 | 4,52E-98 | aquaporin TIP2-1-like |
| Solyc07g040960.1.1 | 2,52E-97 | salt responsive protein 2 [Solanum lycopersicum] |
| Solyc02g089540.2.1 | 1,57E-84 | CONSTANS 1 |
| Solyc12g006050.1.1 | 3,98E-71 | nitrate transporter 1.3-like |
| Solyc08g080190.2.1 | 4,97E-69 | protein HOTHEAD-like |
| Solyc01g109700.2.1 | 7,85E-57 | transcription factor bHLH63-like [Solanum lycopersicum] |
| Solyc09g089510.2.1 | 6,96E-52 | proteinase inhibitor I-B-like |
| Solyc08g082980.2.1 | 9,55E-48 | serine/threonine-protein kinase WNK4-like isoform 1 |
| Solyc06g062460.2.1 | 1,36E-45 | transcription factor bHLH87-like |
| Solyc03g005900.2.1 | 2,66E-45 | GDSL esterase/lipase At5g45670-like |
| Solyc09g084490.2.1 | 4,62E-44 | wound-induced proteinase inhibitor 1-like |
| Solyc11g011030.1.1 | 2,40E-42 | Pto-responsive gene 1 protein |
| Solyc08g077530.2.1 | 2,60E-39 | beta-amylase 3, chloroplastic-like |
| Solyc10g005400.2.1 | 2,76E-39 | inositol oxygenase 1-like |
| Solyc10g007110.2.1 | 2,75E-38 | probable aminotransferase TAT2-like |
| Solyc03g083440.2.1 | 8,53E-38 | glutamate synthase 1 [NADH], chloroplastic-like |
| Solyc05g053410.2.1 | 6,08E-36 | phytochrome B2 |
| Solyc04g009900.2.1 | 2,21E-35 | phosphoenolpyruvate carboxylase kinase |
| Solyc06g060830.2.1 | 1,01E-33 | homeobox-leucine zipper protein HAT1-like |
| Solyc03g114720.2.1 | 2,28E-33 | transcription factor BIM1-like |
| Solyc09g089530.2.1 | 4,09E-32 | wound-induced proteinase inhibitor 1-like |
| Solyc08g079090.2.1 | 6,71E-31 | monocopper oxidase-like protein SKU5-like |
| Solyc12g005300.1.1 | 2,69E-30 | chlorophyllase-2, chloroplastic-like isoform 1 |
| Solyc01g066740.2.1 | 5,07E-28 | putative E3 ubiquitin-protein ligase LIN-like |
| Solyc04g014510.2.1 | 3,02E-27 | glutamine synthetase cytosolic isozyme 1-1 |
| Solyc01g111880.2.1 | 4,41E-24 | tyrosine-protein kinase transforming protein Fes-like |
| Solyc06g006110.2.1 | 5,96E-22 | vacuolar cation/proton exchanger 3-like |
| Solyc10g079320.1.1 | 7,47E-22 | zeatin O-glucosyltransferase-like |
| Solyc01g095150.2.1 | 3,14E-17 | late embryogenesis (Lea)-like protein |
| Solyc06g007190.2.1 | 4,30E-17 | protein phosphatase 2C 2-like |
| Solyc03g093550.1.1 | 5,61E-17 | ethylene-responsive transcription factor 5-like |
| Solyc03g026280.2.1 | 1,80E-16 | CBF1 protein |
| Solyc08g005610.2.1 | 4,20E-16 | abscisic acid 8'-hydroxylase 1-like |
| Solyc01g017090.2.1 | 1,73E-14 | NADH-plastoquinone oxidoreductase subunit 5 |
| Solyc05g005000.2.1 | 1,34E-13 | lipase-like isoform 1 |
