# Supplementary material for: Ectopic Expression of CDF3 Genes in Tomato Enhances Biomass Production and Yield under Salinity Stress Conditions
Source: Front Plant Sci. 2017 May 3;8:660. doi: 10.3389/fpls.2017.00660 (PMC5414387; doi:10.3389/fpls.2017.00660)
Supplement: Supplementary file 6 [file Table6.docx]

| **Table S6*.*** **Effect of the overexpression of *CDF3* gene from either Arabidopsis (line 2.3) or tomato (lines 11.2 and 23.1) on compounds and variables related to organoleptic quality in tomato.** Plants were cultured under control and salinity (75mM NaCl) in the greenhouse from December to mid-August. Four representative fruits, until the 3rd truss, were collected from each plant in the mature-red stage. SSC: soluble sugar content DM: dry matter (%); NT: not transformed. For each growing condition, different letters indicate significant differences (P<0.05). The P value of the ANOVA analysis is provided in the lower part of the Table. ns: not significant. FW: fresh weight. | | | | | | | | | | | | | | | | | | |
| --- | --- | --- | --- | --- | --- | --- | --- | --- | --- | --- | --- | --- | --- | --- | --- | --- | --- | --- |
| Growing conditions | Line | SSC | DM | | Malic acid | | Citric acid (C) | | Glutamic acid (G) | | GABA | Fructose | Glucose | | Sucrose eq. (SE) | | SE/C ratio | SE/G ratio |
|  |  |  |  | | (mg 100 g^-1^ FW) | | | | | | | | | | | |  |  |
| Control | 11.2(Sl) | 4.3 | | 4.8 | | 276 a | | 409 b | | 162 b | 90 ab | 1103 | | 1133 ab | | 2765 ab | 6.8 a | 17.4 a |
|  | 23.1(Sl) | 4.2 | | 4.8 | | 283 a | | 357 b | | 161 b | 97 a | 1170 | | 1191 a | | 2915 a | 8.2 a | 18.5 a |
|  | 2.3(At) | 4.3 | | 4.8 | | 246 a | | 401 b | | 198 b | 82 ab | 1078 | | 1095 ab | | 2682 ab | 7.1 a | 14.8 a |
|  | NT | 4.2 ns | | 5.2 ns | | 150 b | | 691 a | | 227 a | 60 b | 1015 ns | | 884 b | | 2270 b | 3.4 b | 10.7 b |
| 75 mM NaCl | 11.2 (Sl) | 5.4 | | 6.2 | | 302 a | | 558 ab | | 201 ab | 139 a | 1441 a | | 1279 a | | 3265 a | 5.9 b | 18.2 ab |
|  | 23.1 (Sl) | 5.3 | | 6.2 | | 325 a | | 518 bc | | 231 a | 155 a | 1467 a | | 1288 a | | 3299 a | 6.5 b | 14.7 bc |
|  | 2.3 (At) | 4.7 | | 5.3 | | 222 b | | 376 c | | 144 b | 85 b | 1463 a | | 1198 ab | | 3140 ab | 8.4 a | 22.0 a |
|  | NT | 5.2 ns | | 6.1 ns | | 156 c | | 744 a | | 244 a | 72 b | 1129 b | | 1100 b | | 2728 b | 3.8 c | 12.0 c |
| Factor | |  |  | |  | |  | | ***ANOVA analysis*** | | |  |  | |  | |  |  |
| Salinity |  | *<10^-2^* | *<10^-2^* | | *0.41* | | *0.03* | | *0.20* | | *<10^-2^* | *<10^-2^* | *0.03* | | *<10^-2^* | | *0.73* | *0.48* |
| Line |  | *0.53* | *0.09* | | *<10^-2^* | | *<10^-2^* | | *<10^-2^* | | *<10^-2^* | *0.06* | *<10^-2^* | | *0.01* | | *<10^-2^* | *<10^-2^* |
| Salinity x Line | | *ns* | *ns* | | *ns* | | *ns* | | *ns* | | *0.03* | *ns* | *ns* | | *ns* | | *0.03* | *ns* |
