# Supplementary material for: Ectopic Expression of CDF3 Genes in Tomato Enhances Biomass Production and Yield under Salinity Stress Conditions
Source: Front Plant Sci. 2017 May 3;8:660. doi: 10.3389/fpls.2017.00660 (PMC5414387; doi:10.3389/fpls.2017.00660)
Supplement: Supplementary file 7 [file Table7.docx]

| **Table S7. Effect of salinity (75 mM NaCl) on the sodium, sulphur, phosphorous, calcium, magnesium and potassium content (% DW) in leaves, stem and roots of Moneymaker tomato (NT) and transformed plants overexpressing the *AtCDF3* gene (line 2.3)**. Thirty-day-old plants grown in hydroponic culture were subjected to moderate salinity (75 mM NaCl). Mineral elements determinations were performed after 15 days. Each measure is the mean of three independent determinations (after 24 days in stress conditions) in 9 different plants. | | | | | | | | | | | | | | |  |
| --- | --- | --- | --- | --- | --- | --- | --- | --- | --- | --- | --- | --- | --- | --- | --- |
|  |  |  | Na | | S | | P | | Ca | | Mg | | K | | |
| NT | Leaves | Control  Salinity | 0.05  2.40 | b  a | 0.99  0.74 | a  b | 0.83  0.67 | a  b | 2.37  1.65 | a  b | 0.55  0.37 | a  b | 6.89  4.55 | a  b | |
|  | Stem | Control  Salinity | 0.29  4.65 | b  a | 0.61  0.41 | a  b | 0.88  0.76 | ns | 0.97  0.39 | a  b | 0.48  0.32 | a  b | 5.87  3.62 | a  b | |
|  | Roots | Control  Salinity | 0.05  3.64 | b  a | 0.32  0.23 | a  b | 0.48  0.42 | a  b | 1.49  0.98 | a  b | 0.39  0.20 | a  b | 10.32  7.55 | a  b | |
| 35S::AtCDF3 | Leaves | Control  Salinity | 0.05  2.30 | b  a | 0.96  0.86 | ns | 0.87  0.80 | ns | 2.43  2.29 | ns | 0.58  0.50 | ns | 6.78  5.11 | a  b | |
|  | Stem | Control  Salinity | 0.18  3.85 | a  b | 0.52  0.36 | a  b | 0.90  0.68 | a  b | 0.72  0.38 | a  b | 0.39  0.26 | a  b | 6.35  4.09 | a  b | |
|  | Roots | Control  Salinity | 0.05  3.52 | a  b | 0.27  0.24 | ns | 0.49  0.52 | ns | 1.38  1.06 | a  b | 0.36  0.22 | a  b | 10.59  8.21 | a  b | |
| For each genotype, organ and mineral element, different letters indicate significant differences (P<0.05). | | | | | | | | | | | | | | |  |
