# Supplementary material for: Ectopic Expression of CDF3 Genes in Tomato Enhances Biomass Production and Yield under Salinity Stress Conditions
Source: Front Plant Sci. 2017 May 3;8:660. doi: 10.3389/fpls.2017.00660 (PMC5414387; doi:10.3389/fpls.2017.00660)
Supplement: Supplementary file 8 [file Image1.PDF]

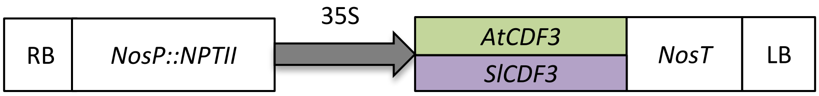

Figure S1. Schematic representation of the construction used for tomato transformation. The construction contains the ORF of the corresponding gene (*AtCDF3* or *SlCDF3*), flanked by the CaMV35S promoter and the nopaline synthase gene (NOS) 3´terminator. The *NPTII* gene under the control of the NOS promoter was used as a selective marker.
