# Supplementary material for: Ectopic Expression of CDF3 Genes in Tomato Enhances Biomass Production and Yield under Salinity Stress Conditions
Source: Front Plant Sci. 2017 May 3;8:660. doi: 10.3389/fpls.2017.00660 (PMC5414387; doi:10.3389/fpls.2017.00660)
Supplement: Supplementary file 9 [file Image2.PDF]

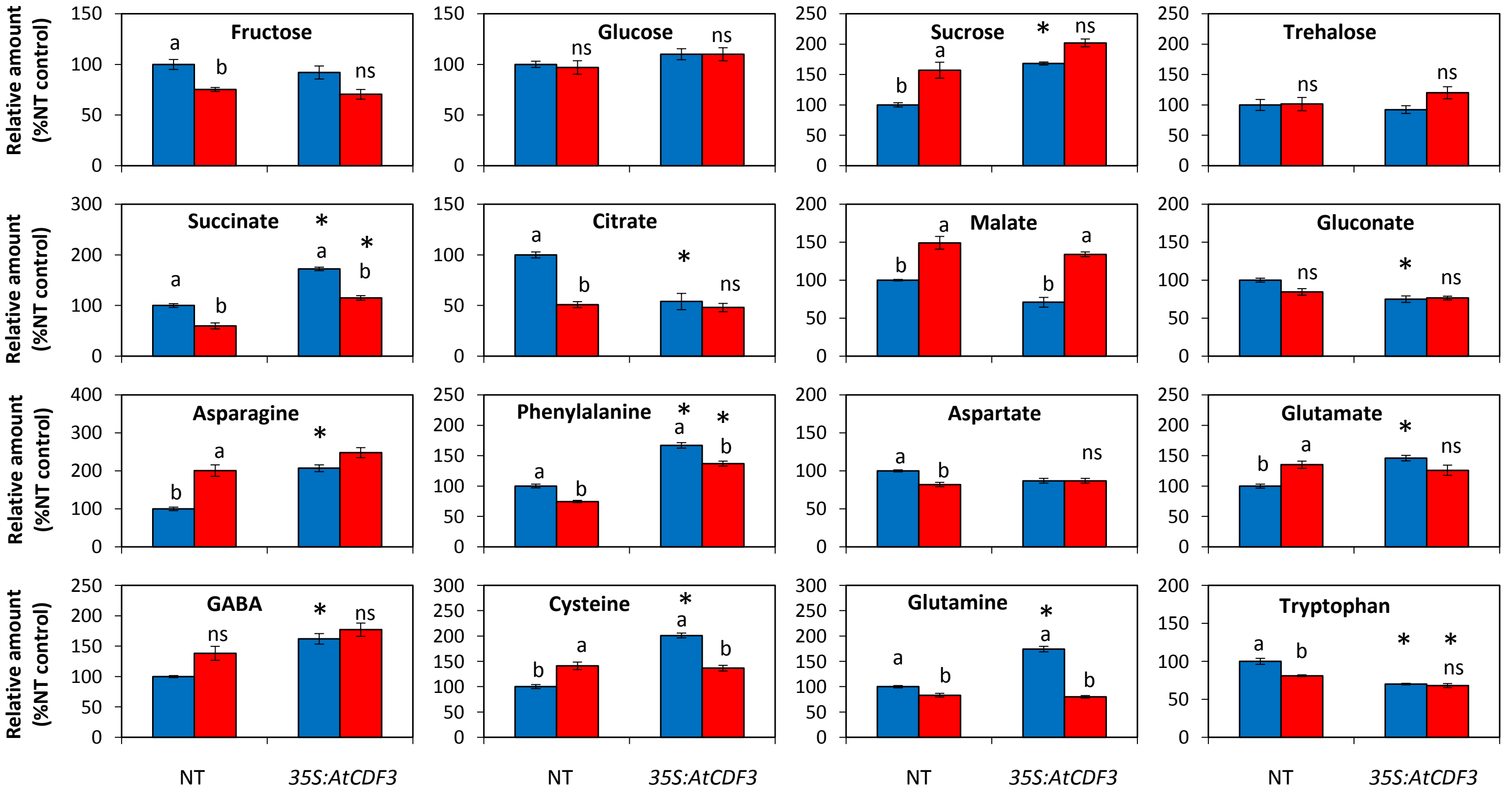

Figure S2. Metabolite analysis of the non-transformed (NT) and *35S::AtCDF3* (line 2.3) plants under control and salinity conditions. Similar results were obtained for line 10.1 (data not shown). Thirty-day-old NT and *35S::AtCDF3* plants were grown in hydroponic culture under control (blue bars) and salinity (75 mM NaCl, red bars) conditions. Metabolomic determinations in leaves were performed after 15 days. Relative quantities (% of NT) of the selected metabolites analysed by gas chromatography-selected ion monitoring-mass spectrometry. Means±SE (n=15) are provided. Similar results were obtained in five independent experiments. Different letters indicate significant differences ($P<0.05$) in each genotype for the stress effect. Differences between genotypes per treatment are indicated by an asterisk.
