# Supplementary material for: Ectopic Expression of CDF3 Genes in Tomato Enhances Biomass Production and Yield under Salinity Stress Conditions
Source: Front Plant Sci. 2017 May 3;8:660. doi: 10.3389/fpls.2017.00660 (PMC5414387; doi:10.3389/fpls.2017.00660)
Supplement: Supplementary file 10 [file Image3.PDF]

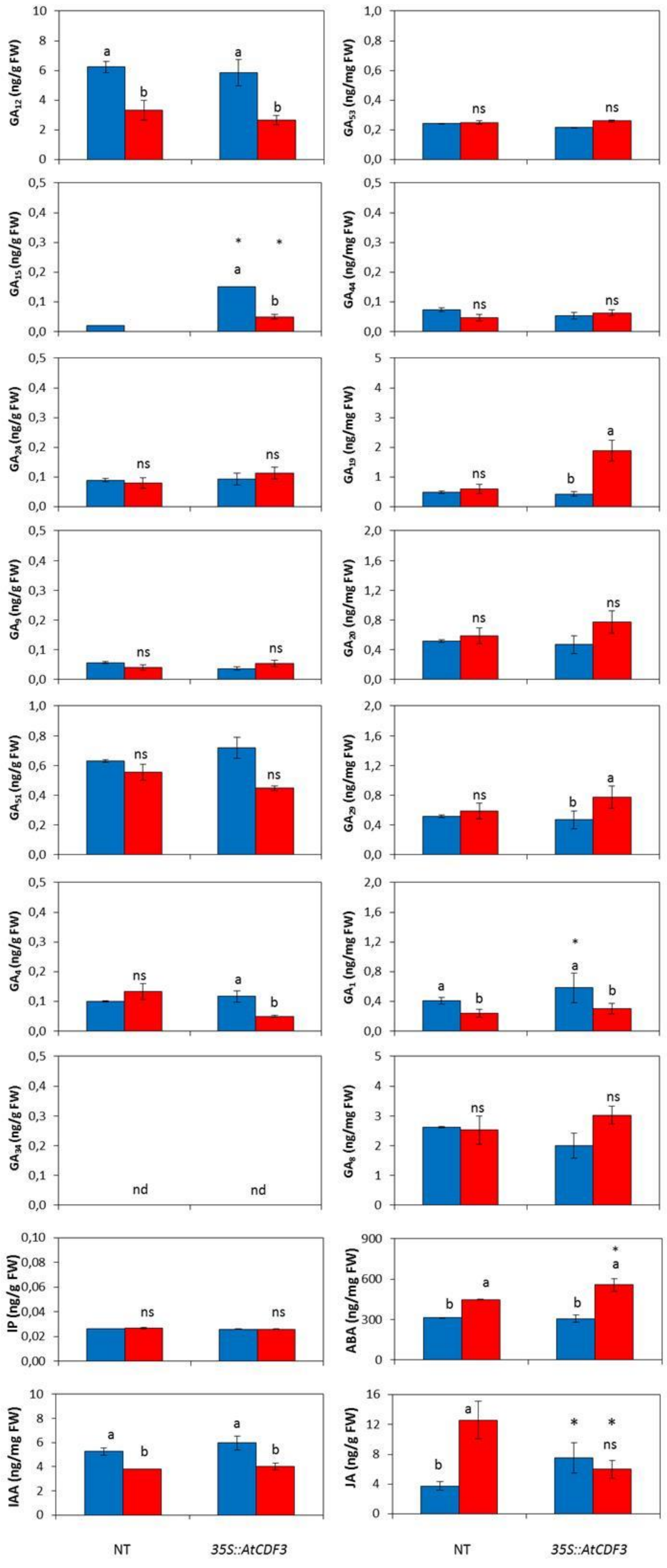

Figure S3. **Effect of the overexpression of *AtCDF3* gene on hormone content in leaves.** The gibberellins (GA_{12}, GA_{15}, GA_{24}, GA_9, GA_{51}, GA_4, GA_{34}, GA_{53}, GA_{44}, GA_{19}, GA_{20}, GA_{29}, GA_1, GA_8), auxin (IAA), cytokinin (IP), jasmonates (JA) and ABA levels in leaves were shown in the NT and *35S::AtCDF3* plants (line 2.3). Thirty-day-old plants were grown in hydroponic culture under control (blue bars) and salinity (75 mM NaCl, red bars) conditions. Metabolomic determinations in leaves were performed after 15 days. Different letters indicate the significant differences ($P<0.05$) within each genotype for the stress effect. Differences between genotypes per treatment are indicated by an asterisk.
